# Zytux in Refractory Myasthenia Gravis: A Multicenter, Open-Labeled, Clinical Trial Study of Effectiveness and Safety of a Rituximab Biosimilar

**DOI:** 10.3389/fneur.2021.682622

**Published:** 2021-08-26

**Authors:** Farzad Fatehi, Kamyar Moradi, Ali Asghar Okhovat, Ghazaleh Shojatalab, Behnaz Sedighi, Reza Boostani, Payam Sarraf, Bahram Haghi Ashtiani, Majid Ghasemi, Soussan Moussavi, Nassim Anjidani, Shahriar Nafissi

**Affiliations:** ^1^Neurology Department, Shariati Hospital, Tehran University of Medical Sciences, Tehran, Iran; ^2^Neurology Department, Sina Hospital, Tehran University of Medical Sciences, Tehran, Iran; ^3^Neurology Department, Shafa Hospital, Kerman University of Medical Sciences, Kerman, Iran; ^4^Neurology Department, Ghaem Hospital, Mashhad University of Medical Sciences, Mashhad, Iran; ^5^Neurology Department, Imam-Khomeini Hospital Complex, Tehran University of Medical Sciences, Tehran, Iran; ^6^Iranian Center of Neurological Research, Tehran University of Medical Sciences, Tehran, Iran; ^7^Department of Neurology, Firoozgar Hospital, Iran University of Medical Sciences, Tehran, Iran; ^8^Department of Neurology, Kashani Hospital, Isfahan University of Medical Sciences, Isfahan, Iran; ^9^Medical Student, Faculty of Medicine, Mashhad University of Medical Sciences, Mashhad, Iran; ^10^Head of Medical Department, Orchid Pharmed Company, Tehran, Iran

**Keywords:** clinical response, myasthenia gravis, refractory, rituximab, zytux TM

## Abstract

**Objectives:** Myasthenia gravis (MG) is an immune-mediated neuromuscular disorder responsive to immunomodulatory treatments. 10–20% of MGs are not responsive to conventional first-line therapies. Here, we sought to investigate the efficacy and safety of rituximab therapy in the treatment of patients with refractory MG.

**Methods:** In a 48-week, multicenter, open-labeled, prospective cohort setting, 34 participants with refractory MG were assigned to receive infusions of Zytux, which is a rituximab biosimilar, according to a validated protocol. Clinical, functional, and quality of life (QoL) measurements were recorded at baseline, and seven further visits using the Myasthenia Gravis Foundation of America (MGFA), Myasthenia Gravis Composite (MGC), Myasthenia Gravis Activities of Daily Living profile (MG-ADL), and Myasthenia Gravis Quality of Life (MGQoL-15) scales. Besides, the post-infusion side effects were systematically assessed throughout the study.

**Results:** The correlation analysis performed by generalized estimating equations analysis represented a significant reduction of MGC, MG-ADL, and MGQoL-15 scores across the trial period. The subgroup analysis based on the patients' clinical status indicated a significant effect for the interaction between time and MGFA subtypes on MG-ADL score, MGC score, and pyridostigmine prednisolone dose, reflecting that the worse clinical condition was associated with a better response to rituximab. Finally, no serious adverse event was documented.

**Conclusions:** Rituximab therapy could improve clinical, functional, and QoL in patients with refractory MG in a safe setting. Further investigations with larger sample size and a more extended follow-up period are warranted to confirm this finding.

**Clinical Trial Registration:** The study was registered by the Iranian Registry of Clinical Trials (IRCT) (Code No: IRCT20150303021315N18).

## Introduction

Myasthenia gravis (MG) is a neuromuscular disorder that is caused by an antibody-mediated autoimmune reaction to post-synaptic proteins of the neuromuscular junction (1). MG symptoms include fluctuating fatigable muscle weakness that, in the beginning, commonly occurs in eye muscles but may become generalized and affect respiratory, bulbar, and limb-girdle muscles (2, 3). Significant improvements in the diagnosis and treatment of MG have led to a higher estimated prevalence but lower mortality rate because of the disease (4). Currently, a variety of pharmacological interventions (i.e., acetylcholine esterase inhibitors, corticosteroids, and non-steroidal immunosuppressive agents) are available for the treatment of MG patients to achieve complete stable remission (5). However, it has been shown that 10–15% of the patients, so-called refractory MG, do not entirely respond to conventional treatments or experience severe side effects related to immunosuppressive medications, most likely attributable to a heterogeneous pattern of the disease pathophysiology leading to diverse disease courses (6, 7).

There is no widely accepted consensus-based definition of refractory MG; operational definitions are often used to establish patient populations for analysis or as entry criteria for clinical studies. The suggested criteria include the following: failure of adequate response to conventional therapies with maximal possible safe doses of corticosteroids and at least one or more immunosuppressive drug at an adequate dose and period; inability to lessen immunosuppressive therapy without clinical relapse or a need for continuing rescue therapy such as intravenous immunoglobulin G (IVIg) or plasma exchange (PLEX); intolerable adverse effects from immunosuppressive therapy; comorbidity that confines the usage of conventional therapies; recurrent myasthenic crises even though the patient is on suitable therapy (4).

Rituximab is a chimeric monoclonal antibody that targets the CD20 antigen of mature B cells and provokes B cell–depleting mechanisms (4). This medication has been approved for several autoimmune conditions (8), such as rheumatoid arthritis (RA) (9), non-Hodgkin's lymphoma (10), and anti-neutrophil cytoplasmic antibody–associated vasculitis (11, 12). Various case reports and retrospective case series have demonstrated favorable efficacy and safety of rituximab in treating refractory MG (13–18). In addition, limited prospective data exist concerning the remarkable therapeutic role of rituximab in refractory MG (19, 20). Taken together, it appears that rituximab might be an appropriate alternative for patients with refractory MG with suboptimal response to the routine approaches, although it requires further confirmation.

In this study, we designed a 48-week, prospective, pre–post trial and explored the efficacy and safety of rituximab in refractory MG treatment with different subcategories of the disorder. We believe that the results of this study could provide novel information regarding the appropriateness of rituximab adjunctive therapy for those suffering from refractory MG.

## Materials and Methods

### Trial Design and Setting

**Figure 1:**
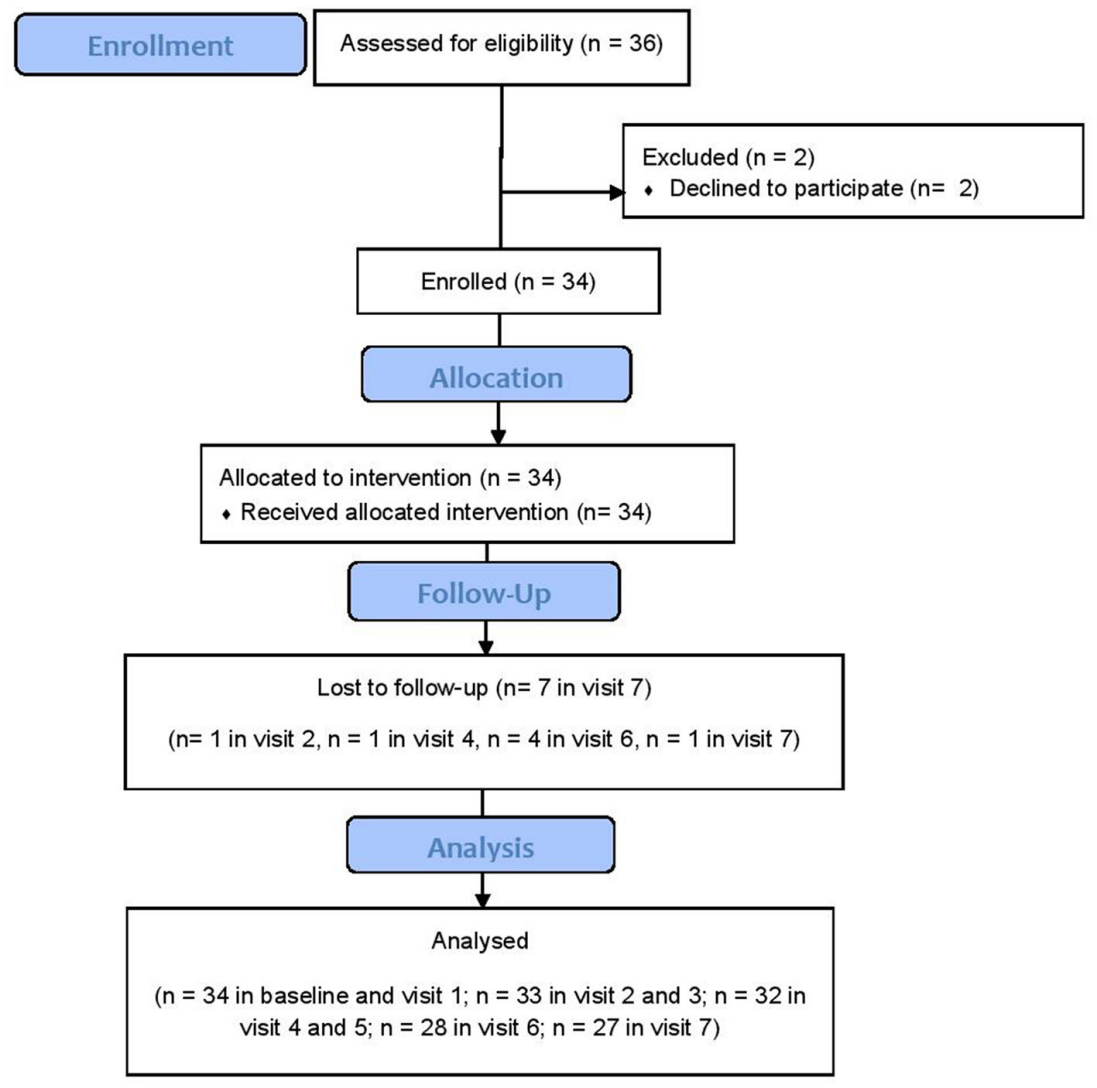
CONSORT flow diagram of the study.

The present study was a 48-week, multicenter, open-labeled, prospective, pretest–posttest trial conducted in five neuromuscular referral centers in Iran (Figure 1). The trial was in accordance with the Declaration of Helsinki and its consecutive revisions (21), and the protocol was approved by the Institutional Review Board (IRB) of Tehran University of Medical Sciences (IR.TUMS.MEDICINE.REC.1398.879). After a complete description of the procedures and purpose of the trial, written informed consent was obtained from the participants. Patients were aware of their right to withdraw from the trial without any negative effect on their standard treatment plan. The trial was registered in the Iranian registry of clinical trials, which is part of the World Health Organization Registry Network (www.irct.ir; trial identifier with the IRCT database: IRCT20150303021315N18).

### Patient Enrollment

A case report form (CRF) was designed to gather patients' information (Appendix 1). This CRF included information about the study design, study schedule from screening day to visit 7, eligibility and exclusion criteria, patients' demographics and contacts, physical examination at each session, concomitant medication use, history of medications use and relapses, history of thymectomy, laboratory data including antibody status (AchR antibody positive, anti-MUSK positive or double seronegative), outcome measurement questionnaire consisting of MG quality-of-life questionnaire (MG-QoL15), Myasthenia Gravis Activities of Daily Living (MG-ADL), Myasthenia Gravis Foundation of America (MGFA) Clinical Classification, Myasthenia Gravis Composite Scale (MGC), rituximab infusion form including infusion protocol, evaluations for rituximab adverse effects such as rituximab infusion reaction, classification of infusion reactions, serious adverse events, and serious adverse events classification.

### Participants

Participants in the present trial were adults aged 18–80 years who were selected from an outpatient neuromuscular center referred with the diagnosis of generalized non-thymomatous MG. The diagnosis was made according to clinical observations, and laboratory evaluations such as positive autoantibodies for either muscle-specific tyrosine kinase (MuSK) or acetylcholine receptor (AChR). Tensilon test, single-fiber EMG, and exclusion of other diagnoses were employed for patients with a probable double-seronegative MG. Recruited subjects must have had refractory MG based on either of the following items: 1) unchanged or worsening of clinical response (defined as no change or no improvement in either MG-ADL or MGC score) to a combination of 1 mg/kg/day prednisolone and cytotoxic medications such as azathioprine, mycophenolate mofetil, cyclosporine, cyclophosphamide, or recue therapy (PLEX or IVIg) for at least 12 months (5); 2) severe side effects related to multiple immunosuppressive therapies. Subjects with a previous history of rituximab therapy were excluded. Other exclusion criteria were a history of allergy to prednisolone, pregnancy, lactation, presence of active infectious disease, history or presence of cancer, hepatitis B, and the presence of several medical conditions (e.g., diabetes mellitus, cardiac arrhythmia, chronic cardiac, liver or kidney problems, anemia, peptic ulcer, gastrointestinal bleeding, hypertension, increased intracranial pressure, and cataract). The CONSORT flow diagram is shown in Figure 1. As evident, two patients refused to participate in the study at baseline, and one patient was lost to follow-up in visit 2, one patient in visit 4, four patients in visit 6, and one patient in visit 7 (total number of dropouts until trial endpoint was 7).

### Intervention

Eligible subjects discontinued cytotoxic treatment while they were allowed to take their other medications over the trial period, including prednisolone and acetylcholinesterase inhibitors (AChEI) regularly. If the patient had a crisis, we did PLEX or IVIg, and if the patient had improved from the crisis, we continued to include the patient in the study. If the patient's crisis did not improve satisfactorily and required other regimens such as monthly IVIg or PLEX or switching to other immunosuppressive medications, we withdrew the patient. Participants received 1,000 mg rituximab (Zytux) intravenously in each treatment cycle. Zytux is a biosimilar medication with the generic name of rituximab. It is a product of AryoGen Pharmed, an Iranian pharmaceutical company established in 2010, aiming to produce biological products. In a study in patients with chronic lymphocytic leukemia, no clinical differences between the two groups were found regarding the effectiveness of Zytux and Mabthera (Rituximab; Roche) (22). Patients received the first dose at the start of the study and another 1 g of the reagent after 2 weeks. The next doses were administered to the patients in intervals of 6 months from the previous cycle.

### Outcome Measurements

All eligible participants were evaluated with a complete physical examination, laboratory tests, and vital signs assessment at baseline and weeks 1, 3, 12, 24, 36, and 48 of the trial period. The outcomes of interest were the alteration in scores of the MGC (23, 24), MGQoL-15 (25), MGFA (26), and MG-ADL (27) scoring systems, as well as prednisolone and pyridostigmine doses. The validated versions of MGQoL-15, MGFA, MGC, and MG-ADL scoring systems were used at baseline and weeks 12, 24, 36, and 48 to assess clinical, functional changes and QoL status in response to the intervention.

### Safety

In each session of the visit, the patients were assessed for safety issues. Patients and their caregivers were asked to report the research coordinates any unexpected symptoms immediately through 24-h accessible phone contact. Laboratory tests, including complete blood counts, serum electrolytes, and liver and kidney function tests, were recommended in all visits except visit 3, and any significant deviation of a laboratory test was managed accordingly. Furthermore, at each visit, we monitored the proper use of concomitant medications, corticosteroids, and AChEIs, any signs of MG crisis needing hospitalization, or either PLEX or IVIg prescription. Moreover, all adverse events due to trial interventions were systematically recorded at each measurement point using a comprehensive checklist of side effects and a questionnaire of severe complications.

### Statistical Analysis

Statistical analyses were conducted using the R Studio software (R version 3.1.2). *P*-values below 0.05 were regarded as significant. Continuous data are presented as mean ± SD. We used the Shapiro–Wilk and probability graphs to check for the normal distribution of the data.

Since we had missing values in longitudinal data analysis, we used the generalized estimating equations (GEE) model to confront this shortcoming. GEE is a general statistical method to fit a marginal model for the analysis of longitudinal/clustered data, generally applied into clinical trials. This approach is a population-level method based on a quasi-likelihood function providing the population-averaged estimations of the parameters (28).

The GEE was carried out to evaluate the alterations in the average of scores on MGC score, MGFA, MG-ADL, MGQoL-15, prednisolone dose, and pyridostigmine dose over the trial period. Besides, participants were subcategorized into two groups based on the MGFA cut-off level. We considered the patients with mild weakness (i.e., MGFA scores of I, IIa, IIb) as low MGFA score and the patients with moderate to severe weakness, i.e., MGFA ≥3, as high MGFA score (14). Afterward, prednisolone and pyridostigmine dose reduction were compared between the subgroups.

## Results

### Clinical and Functional Changes

**Figure 2:**
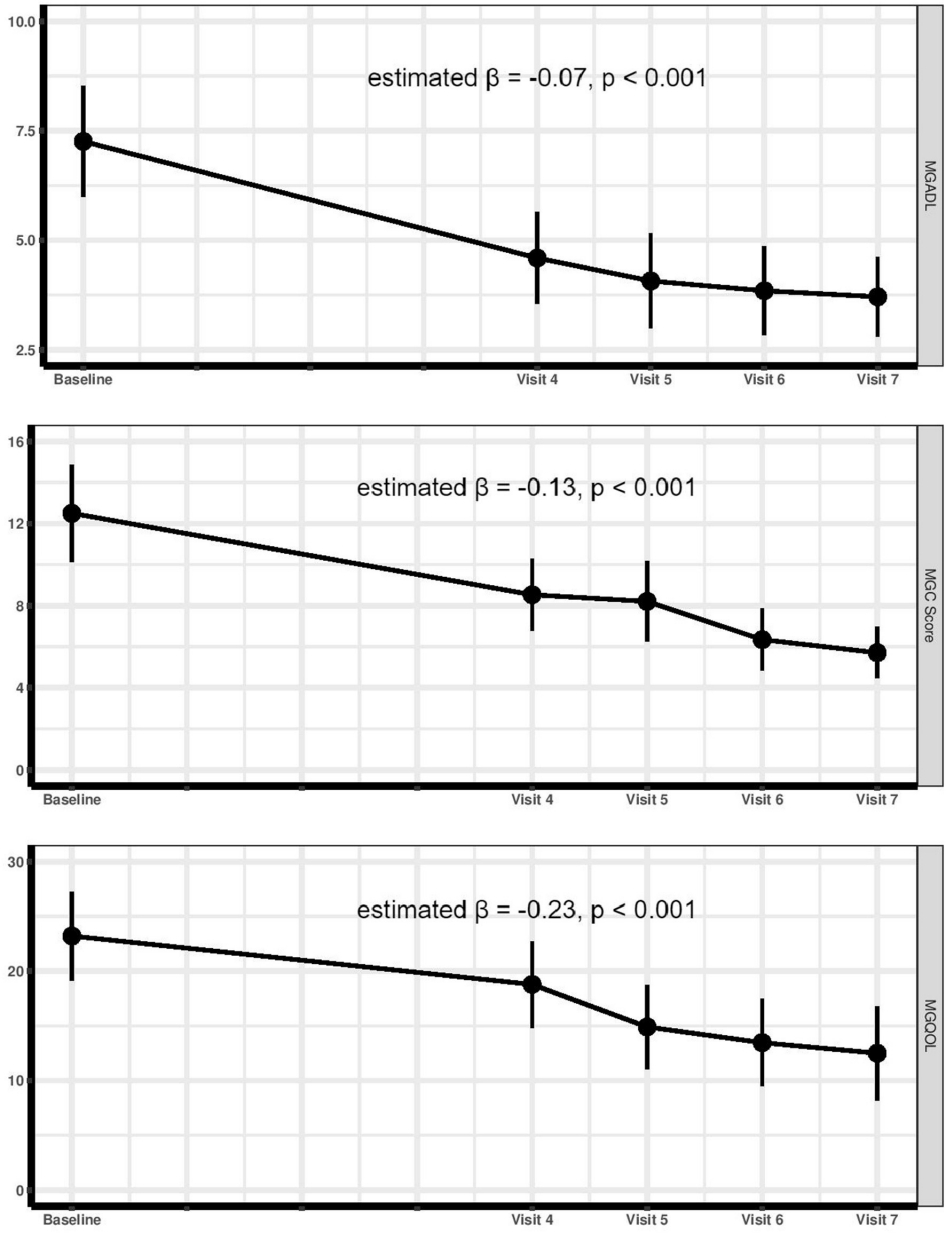
Generalized estimating equations for analyzing the alteration trend of Myasthenia Gravis Activities of Daily Living (MG-ADL), Myasthenia Gravis Composite scale (MGC), and MG Quality of Life Questionnaire (MG-QoL15) scores during the trial period.

**Figure 3:**
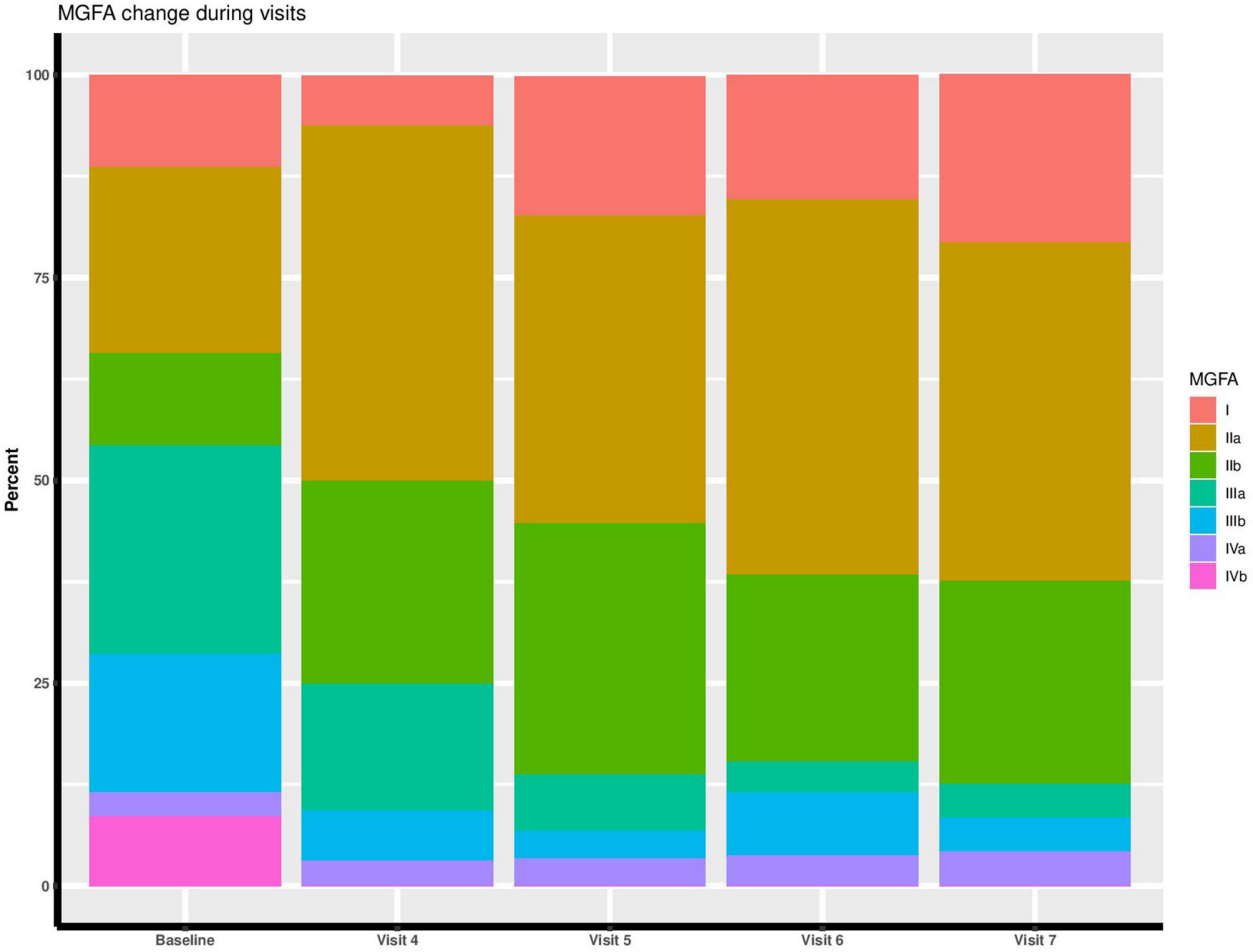
Myasthenia Gravis Foundation of America (MGFA) clinical classification of participants across visits.

The baseline demographic and clinical characteristics of the participants are described in Table 1. Moreover, GEE analysis of clinical and functional quantitative scales across trial visits showed significant reduction of MGC (estimated β = −0.13, *p* < 0.001), MG-ADL (estimated β = −0.07, *p* < 0.001), and MG-QoL15 (estimated β = −0.23, *p* < 0.001) scores (Figure 2). In addition, Figure 3 represents the disease stage in MG subjects within each visit. As evident, according to the MGFA scale, RTX-treated patients experienced improved clinical conditions over time.

**Table 1:** Baseline demographic and clinical characteristics of the participants.

**Age, years, Mean ± SD**	**47.9 ± 15.2**
**Onset age**, years, Mean ± SD	38.0 ± 16.3
**Gender**, Female/Male	22/12
**Subtype**	
AchR+, Number (%)	17
MuSK+, Number (%)	9
Double seronegative, Number (%)	8
**Mean prednisolone dose**, mg/day, Mean ± SD	35 ± 22.3
**Mean pyridostigmine dose**, mg/day, Mean ± SD	235 ± 85
**MGC score**, Mean ± SD	12.5 ± 7.1
**MG-ADL score**, Mean ± SD	7.3 ± 3.8
**MGQoL-15 score**, Mean ± SD	23.2 ± 12.1

### Medication Use Changes

**Figure 4:**
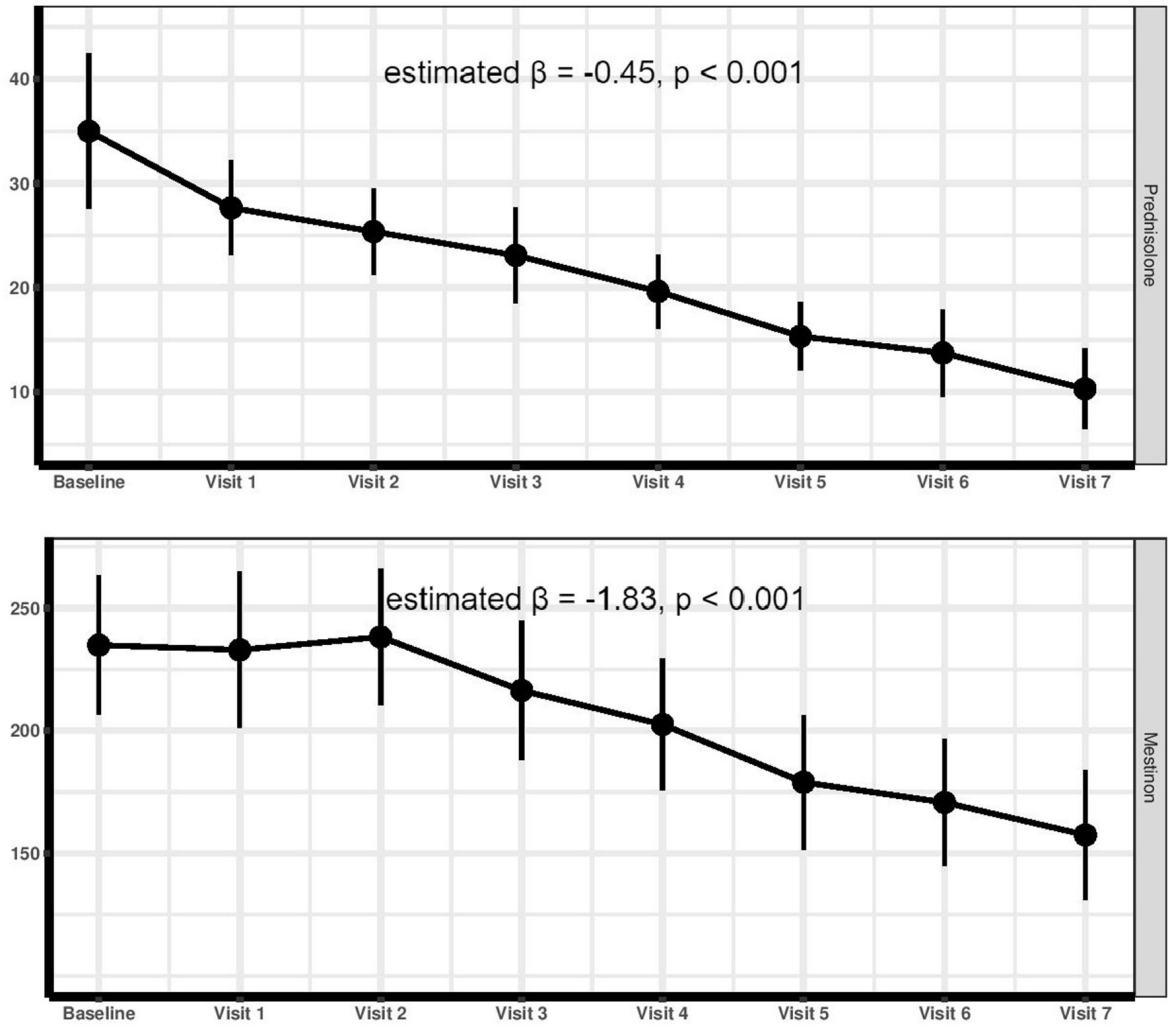
The alteration trend of prednisolone and pyridostigmine administration dose during the trial period.

Interpreting the GEE analysis, a significant dose reduction was observed for both prednisolone (estimated β = −0.45, *p* < 0.001) and pyridostigmine (estimated β = −1.83, *p* < 0.001) during the trial period (Figure 4).

### Relapse

Eight (23.5%) of the patients experienced a clinical relapse during the trial period with a female-to-male ratio of 3:5. Five participants with relapse did not complete the trial (*n* = 1 in visit 4, *n* = 4 in visit 6) due to their need to receive appropriate treatments for their condition (i.e., monthly PLEX or IVIg). Three patients recovered from relapse and, as a result, completed the trial. On the other hand, the antibody status of the mentioned patients was as follows: *n* = 6 for AchR+, *n* = 1 for MuSK+, and *n* = 1 for double seronegative.

### Subgroup Analysis of the Changes Based on Clinical Status

After subdivision of the subjects based on their clinical status at baseline, 16 patients were found to have a stage of below 3 on the MGFA (low MGFA score) (class I: *n* = 4; class IIa: *n* = 8; class IIb: *n* = 4) while 20 participants represented MGFA stage ≥3 (high MGFA score) (class IIIa: *n* = 9; IIIb: *n* = 7; class IVa: *n* = 1; class IVb: *n* = 3).

GEE analysis indicated a significant effect for the interaction between time and MGFA subtype on pyridostigmine dose (estimated β = −1.84, *p* = 0.011), MG-ADL score (estimated β = −0.07, *p* = 0.004), and MGC score (estimated β = −0.11, *p* = 0.002). On another note, however, there was no significant effect for the interaction between time and MGFA subtype on prednisolone dose (estimated β = −0.16, *p* = 0.17) and MGQoL-15 score (estimated β = −0.11, *p* = 0.15). The trend of outcome changes in the subgroups are described in Table 2.

**Table 2:** A summary of outcome changes in each subgroup during the trial period.

**Variable**	**Subgroup**	**Estimated beta**	***P* value**
Mestinon dose	MGFA < 3	−0.65	**< 0.001**
	MGFA ≥ 3	−2.49	**< 0.001**
Prednisolone dose	MGFA < 3	−0.35	**< 0.001**
	MGFA ≥ 3	−0.50	**< 0.001**
MG-ADL score	MGFA < 3	−0.03	0.09
	MGFA ≥ 3	−0.10	**< 0.001**
MGC score	MGFA < 3	−0.05	0.07
	MGFA ≥ 3	−0.17	**< 0.001**
MGQoL-15 score	MGFA < 3	−0.15	**0.014**
	MGFA ≥ 3	−0.26	**< 0.001**

*MGC, Myasthenia gravis composite; MGFA, Myasthenia Gravis Foundation of America; MGQoL-15, Myasthenia Gravis Quality of Life; MG-ADL, Myasthenia Gravis Activities of Daily Living profile. Bold values indicate significant change*.

### Subgroup Analysis of the Changes Based on Antibody Status

Patients were subcategorized into three groups based on the antibody status (MuSK positive, AchR positive, and double seronegative), and the double-seronegative group was selected as the reference in GEE analyses. GEE model exhibited that the presence of antibody for AchR significantly affects pyridostigmine dose (estimated β = 1.82, *p* = 0.013) throughout the study. On another note, antibody positivity for the MuSK receptor influenced pyridostigmine dose (estimated β = 1.53, *p* = 0.011) over the study course. The presence of these two antibodies was shown to exert no significant effect on other outcome measures over the trial period (*p*-values > 0.05). Finally, comparing the two positive antibody conditions (AchR+ and MuSK+), GEE resulted in similar changes over time in terms of all assessed outcomes (*p*-values > 0.05).

### Adverse Events

In the first post-infusion 2 days, mild headache (*n* = 3), flushing (*n* = 1), abdominal pain (*n* = 1), and fever (*n* = 1) were detected. There was no serious or chronic side effect reported by the participants.

## Discussion

In the present study, we demonstrated that rituximab attenuates patients' clinical and functional status with refractory MG. Treated patients exhibited improved symptoms and signs, QoL, and daily functioning. There were significant reductions in the average daily dose of prednisolone and pyridostigmine due to appropriate clinical response and lower rates of relapse. Notably, reduction of pyridostigmine dose, MG-ADL score, and MGC score were found to be more prominent in those who represent severe stages of the disease. Finally, the administration of RTX showed to be well tolerated since there were only mild-to-moderate post-infusion side effects.

The first prescription of RTX for MG returns to 2,000 when a patient developed MG after bone marrow transplantation (29). In a retrospective study of eight patients with refractory MG, Singh and Goyal suggested a considerably beneficial RTX effect as induction therapy (18). Collongues et al. investigated the effect of RTX therapy in 20 patients with either refractory or non-refractory MG and indicated a significant reduction in the annualized relapse rate and the MGFA scores (14). The results were supported by Choi and colleagues in a study with a similar design (30). There have also been a few prospective studies confirming the beneficial effects of RTX in refractory MG management. Landon-Cardinal et al. (31) enrolled 12 refractory generalized AChR+ MG patients that received 1 g of RTX at day 0, day 14, and 6-month follow-up. MG Foundation of America Postintervention Status (MGFA-PIS) had improved in 55% of patients at 12 months. A preliminary investigation of RTX efficacy in six severe, non-responder MG patients reported sustained response to the treatment (32). Later, in another study, a long-term follow-up of 17 patients with refractory MG by Díaz-Manera and colleagues led to the detection of significant improvements in MGFA scores after rituximab (16). Moreover, alleviation in involved muscles' function was observed in two different prospective studies, using the manual muscle testing (MMT) scoring system (20).

Beyond clinical status, in the current investigation, patients were assessed for functional outcomes. There are limited data regarding the changes in QoL of patients with MG following the treatment with RTX infusions. Peres et al. measured QoL in six non-refractory patients using a generic (EQ-5D-3L) and a disease-specific (MG-QoL15) questionnaire and found significant improvements (33). Evaluating QoL in the refractory MG context, a recent study of eight subjects showed no significant changes in the scores of MG-QoL15 following RTX therapy (34). In contrast, we observed a remarkable improvement in QoL after the treatment. The controversy could be justified by the larger sample size of this study and the different types of interventions applied.

Disability interfering with functioning during normal daily activities has been previously reported in refractory MG, and several medications have been trialed in this regard (35). Particularly, low-dose RTX has exhibited beneficial effects in daily functioning management, reflected by the significant reduction in the MG-ADL score at 3 months and 6 months post-intervention (34). The MGC is another validated instrument that is used to cover functional domains affected by MG. MGC has been used in several trials of MG as an outcome measure (36–38). As far as we know, however, there is no study evaluating the alterations in MGC score following treatment with rituximab, and this trial is considered the first of its kind.

In this study, we demonstrated that refractory subjects with severe symptoms benefit more prominently than those in a better clinical state. This novel issue suggests that the beneficial role of RTX therapy might be best defined for the end stages of MG. Further studies seem mandatory to test this hypothesis as it could help achieve the most therapeutic benefit of RTX and improve the design of future trials.

There have been several studies of the efficacy of rituximab depending on the type of antibody, which generally indicates greater efficacy in MuSK + MG. Topakian et al. (39) retrospectively reported the efficacy of RTX in 39 AchR+ and 14 MuSK+ MG patients. Remission was more frequent in patients with MuSK+ vs. those with AchR+ (71.4 vs. 35.9%). However, they noted that the lower response rate in the AchR+ group might be due to a selection bias of severely affected patients with longstanding refractory disease. In another study, 14 patients with refractory MG (6 MuSK+, 5 AChR +) were treated with RTX prospectively. All patients responded dramatically to RTX, as measured by a change in MMT score, prednisone dose, or the frequency of IVIg infusions or PLEX (19). Based on the literature, patients who are positive for AchR or MUSK antibody could exhibit an even higher clinical response to RTX (20, 40, 41). In our patients, both AchR+ and MuSK+ MG patients had appropriate therapeutic responses. Additional studies are warranted to clarify this issue.

Herein, we indicated that the positivity of either AchR or MuSK antibody results in a higher dose of pyridostigmine needed over the trial period. It could be interpreted from this finding that patients with AchR+ or MuSK+ antibody are more resistant to treatments and experience less clinical response to RTX therapy. However, this hypothesis is not confirmed, as there was no reduced clinical response according to other measures.

In this study, we discontinued cytotoxic medications at the start of the study to prevent possible immunosuppressive side effects that may emerge from the combined use of rituximab and cytotoxic medications; however, this approach may increase the possibility of relapses in the short term, and it may be advisable to taper cytotoxic medications gradually.

In brief, it seems that rituximab, as an add-on therapy, could alleviate disease severity in patients who do not change or worsen after a combination of prednisolone and cytotoxic medications, or rescue therapy or severe side effects related to multiple immunosuppressive therapies. Moreover, both groups of patients with AchR+ or MuSK+ antibody may benefit from rituximab.

Some limitations should be considered. First, the self-controlled open-label design and limited follow-up time might have influenced the results. Second, further studies of different doses and regimens should be considered different RTX dosages that may have different therapeutic effects. Third, the limited observation time, and the relatively small sample size with a significant dropout rate, may restrict the generalizability of the results. Fourth, this trial included no control group to ascertain the intervention effect. Additional clinical trials with a parallel-arm, placebo-controlled design are required for this purpose. Finally, although there is no strict RTX dosage protocol in the treatment of MG, the levels of circulating CD19^+^ and CD20^+^ B cells could be an appropriate marker for determining the need for RTX re-infusions.

## Conclusion

RTX add-on to prednisolone appeared to be safe and well tolerated in patients with refractory MG. Meaningful beneficial effects of the treatment strategy were reflected from the validated tools measuring the patients' clinical, functional, and QoL. In support, dose reduction of prednisolone and pyridostigmine was observed in the participants. On another note, we showed that patients with severe clinical conditions benefit from the RTX intervention more prominently. To our knowledge, this multicenter trial is currently considered one of the largest, evaluating the efficacy and safety of RTX in refractory MG. However, further studies are warranted to confirm our findings.

## Data Availability Statement

The raw data supporting the conclusions of this article will be made available by the authors, without undue reservation.

## Ethics Statement

The studies involving human participants were reviewed and approved by Iranian Registry of Clinical Trials (IRCT), which is part of the World Health Organization Registry Network (Code No: IRCT20150303021315N18). The patients/participants provided their written informed consent to participate in this study.

## Author Contributions

FF designed and conceptualized the study, analyzed the data, and drafted the article for intellectual content. KM drafted the article for intellectual content. AO gathering and examining the patients and drafting and revision for intellectual content. GS, BS, RB, PS, BH, MG, and SM gathering and examining the patients. NA designed and conceptualized study and analyzed the data. SN led and coordinated communication among sites, designed and conceptualized study, and drafted the article for intellectual content. All authors contributed to the article and approved the submitted version.

## Conflict of Interest

NA was employed by company Orchid Pharmed. The remaining authors declare that the research was conducted in the absence of any commercial or financial relationships that could be construed as a potential conflict of interest. The authors declare that this study received funding from Aryogen Pharmaceutical Company. The funder had the following involvement in the study: participating in the design including case report forms (CRFs), conceptualization, and analysis of the study.

## Publisher's Note

All claims expressed in this article are solely those of the authors and do not necessarily represent those of their affiliated organizations, or those of the publisher, the editors and the reviewers. Any product that may be evaluated in this article, or claim that may be made by its manufacturer, is not guaranteed or endorsed by the publisher.
