# Supplementary material for: Zytux in Refractory Myasthenia Gravis: A Multicenter, Open-Labeled, Clinical Trial Study of Effectiveness and Safety of a Rituximab Biosimilar
Source: Front Neurol. 2021 Aug 26;12:682622. doi: 10.3389/fneur.2021.682622 (PMC8427762; doi:10.3389/fneur.2021.682622)
Supplement: Supplementary file 1 [file Presentation_1.pdf]

CASE REPORT FORM

Evaluation of efficacy and safety of Zytux™ (Rituximab) in refractory myasthenia gravis patients

This trial is a pre-post study, in which, patients diagnosed with myasthenia gravis and have not responded to combination of azathioprine and corticosteroids, are considered as refractory myasthenia gravis patients and are eligible to participate in this trial. Azathioprine will be discontinued, but patients can receive corticosteroids and/or pyridostigmine during the trial. The therapeutic schema is rituximab 1000 mg, 2 times (at day 0 and 15), followed by one single injection (1000 mg) 6 months latter. However, repeat rituximab infusions will be administered sooner if myasthenic symptoms reappear and interfere with daily life activities of patient (the interval between Rituximab infusions should be at least three months).
In this study, patients will be compared with themselves, before and after taking Rituximab.

Sponsor: Aryogen Pharmed

Address: No. 140. Corner of Tajbakhsh St., 24th Km Tehran-Karaj Makhsous Road, Alborz, Iran

Tel: +98 26 36106480-4

Fax: +98 26 36104644

Principle Investigator:

Dr. Shahriar Nafissi

Department of Neurology, Shariati Hospital, Tehran University of Medical Sciences

Tel:+98 21 8490 2224

Fax: +98 21 2288 4420

E-mail: nafisi@sina.tums.ac.ir

STUDY SCHEDULE

Tests and assessment	Screening Day -10 to 0	Visit1 (wk1)	Visit2 (wk3)	Visit3 (wk7)	Visit4 (wk12)	Visit5 (wk24)	Visit6 (wk36)	Visit7 (wk48)
**Demographic**	√							
**Medical history**	√							
**Vital signs**	√	√	√		√	√	√	√
**Physical examination**	√	√	√		√	√	√	√
**Concomitant medication**	√	√	√		√	√	√	√
**Relapse history**	√							
**MG Composite**	√				√	√	√	√
**MGQOL15**	√				√	√	√	√
**MG-ADL**	√				√	√	√	√

Date (dd/mm/yy): |___|___|/|___|___|/|___|___|

Patient name/Surname: ……………… Study Site: ………………

Tests and assessment	Screening Day -10 to 0	Visit1 (wk1)	Visit2 (wk3)	Visit3 (wk7)	Visit4 (wk12)	Visit5 (wk24)	Visit6 (wk36)	Visit7 (wk48)
**MGFA**	√				√	√	√	√
**MGFA Post-intervention Status**						√		√
**Eligibility criteria**	√							
**Participant eligibility review**	√							
**Relapse record after treatment**		√	√		√	√	√	√
**Lab data**	√	√	√		√	√	√	√
**Evaluation of CD19 and CD20**				√		√		
**Evaluation of Anti-Musk ab and Anti-AchR ab**	√							√
**Change in corticosteroids dosage**		√	√		√	√	√	√
**Change in pyridostigmine dosage**		√	√		√	√	√	√
**Evaluation for IVIG and plasmapheresis**	√	√	√		√	√	√	√

Patient name/Surname: ………………. Study Site: ……………….

Tests and assessment	Screening Day -10 to 0	Visit1 (wk1)	Visit2 (wk3)	Visit3 (wk7)	Visit4 (wk12)	Visit5 (wk24)	Visit6 (wk36)	Visit7 (wk48)
**Infusion form**		√	√			√		
**Infusion reaction form**		√	√			√		
**SAE**		√	√		√	√	√	√
**AE check list**		√	√		√	√	√	√
**Adverse event**		√	√		√	√	√	√
**Subject off study**		√	√		√	√	√	
**Standard visit sheet**		√	√		√	√	√	√
**PI sign off**	√							√

Date (dd/mm/yy): |___|___|/|___|___|/|___|___|

SCREENING DAY -10 to 0

DEMOGRAPHICS

Patient name/Surname: ………………

Birthdate: ----/--/-- (year/month/day)   □ Approximate □ Unavailable

Gender: □ Male   □ Female

Weight: -------   Height: -------------

Contact information

Address:	
City:	
Phone number: -------------------------<br>□ home □ work □ cell □ other	Alternate phone number: -------------------------<br>□ home □ work □ cell □ other
Preferred method of contact:	
E-mail address:	

Emergency contact

Name:	
Address:	
City:	
Phone number: -------------------------<br>□ home □ work □ cell □ other	Alternate phone number: -------------------------<br>□ home □ work □ cell □ other
Preferred method of contact:	
E-mail address:	

SCREENING DAY -10 to 0

MEDICAL HISTORY

Examine the following and place a √ in the appropriate column. If abnormal is √, then provide the condition(s) in the comments column as provided.

Body system	Normal	Abnormal	Note done	comments
Body as a whole				
HEENT				
Cardiovascular				
Respiratory				
Gastrointestinal				
Genitourinary				
Musculoskeletal				
Neurological				
Endocrinological				
Dermatologic/skin				
Hematologic/Lymphatic				
Metabolic				
Nutritional				
Allergy/ Drug sensitivity				
Psychiatric				

SCREENING DAY -10 to 0

VITAL SIGNS	
Temperature:<br><br>Respiration rate:	Blood Pressure:<br><br>Heart Rate:

PHYSICAL EXAMINATION

Examine the following and place a √ in the appropriate column. If abnormal is √, then provide the condition(s) in the comments column as provided.

Body system	Normal	Abnormal	Not done	comments
Appearance				
Skin				
HEENT				
Thyroid				
Chest				
Renal system				
Cardiovascular system				
Breasts				
Heart				
Abdomen				
Musculoskeletal				
Genitalia				
Pelvic				
Rectal				
Prostate				
Vascular				
Neurological				
Lymph nodes				
specify, others				

Date (dd/mm/yy): |___|___|/|___|___|/|___|___|

SCREENING DAY -10 to 0

CONCOMITANT MEDICATION

Has the participant used any Concomitant Medications? **No □ Yes □,** Complete below

CM No.	Medication name (Record <specify Generic or Brand> name)	Start date (year/month/day)	Stop date (year/month/day)	Or tick if ongoing at end of study?	Reason for use (Enter related AE diagnosis, or other reasons for use, e.g. Prophylaxis)	Dose (Units)	Route	Frequency
1								
2								
3								
4								
5								
6								
7								
8								
9								
10								
11								
12								
13								
14								
15								
16								

Date (dd/mm/yy): |___|___|/|___|___|/|___|___|

SCREENING DAY -10 to 0

RELAPSE HISTORY FORM

Please mention all of the patient relapses before treatment. And mention the first relapse.

Relapse	Date	Ocular Myasthenia Gravis	Generalized Myasthenia Gravis	Duration of relapse	Treatment

IVIG and Plasmapheresis history:

Has patient received IVIG during the last year or least 6 months before inclusion in the study?

No □ Yes □

If yes, please mention the dose and number of administrations:

Has patient undertaken plasmapheresis during the last year or least 6 months before inclusion in the study?

No □ Yes □

If yes, please mention the number of this operation:

SCREENING DAY -10 to 0

ELIGIBILITY CRITERIA

Inclusion criteria

Patients who meet *all* of the following criteria are eligible for enrollment as study participants:

	Yes	No
1. GENERALIZED NON-THYMOMATOUS mg PATIENTS INCLUDING:<br>• *MUSK ANTIBODY POSITIVE*<br>• *ACHR ANTIBODY POSITIVE*<br>• *SERONEGATIVE MYASTHENIA GRAVIS PATIENTS*		
2. Not having previous treatment with ritiuximab		
3. REFRACTORY TO STANDARD TREATMENT OR CONTRAINDICATED INCLUDING:<br>a. CORTICOSTEROID DEPENDENCY:<br>• TAKING AT LEAST 2 MONTHS OF 1MG/KG CORTICOSTEROID MEDICATIONS AND HAS NOT RESPOND PROPERLY<br>• OR, BEING STABLE WITH STEROID THERAPY, BUT DURING TAPERING DOWN THE MEDICATION UP TO 30 MG ALTERNATIVE OR 15 MG DAILY, HAS EXPERIENCED RELAPSE OF THE DISEASE.<br>b. HAVING CONTRAINDICATION OF CORTICOSTEROID THERAPY<br>c. NOT RESPONDER TO AZATHIOPRINE (2-3 MG/KG) THERAPY FOR AT LEAST 9 MONTHS OF TAKING THE MEDICATION OR BECAUSE OF OCCURRENCE OF ADVERSE EFFECTS OF AZATHIOPRINE, THE MEDICATION HAS BEEN STOPPED		
4. $18 \leq Age \leq 80$		

EXCLUSION CRITERIA

PATIENTS WHO MEET *ANY* OF THESE CRITERIA ARE *NOT* ELIGIBLE FOR ENROLLMENT AS STUDY PARTICIPANTS:

	YES	NO
**PREGNANCY**		
**PLANNING FOR PREGNANCY IN THE FOLLOWING YEAR**		
**LACTATION**		
**ACTIVE INFECTIONS**		
**HBsAg+ OR HBV DNA POSITIVE**		
**PRIOR MALIGNANCY**		
**HYPERSENSITIVITY REACTIONS OR ANAPHYLACTIC SHOCK TO STUDIED DRUGS**		
**MAJOR RENAL DISEASE**		
**MAJOR HEPATIC DISEASE**		
**CARDIAC ARRHYTHMIA HISTORY**		
**OCULAR MG**		
**THYMOMATOUS MG**		
**MAJOR PSYCHIATRIC DISEASE**		

<u>EXCLUSION FROM THE STUDY:</u>

- **Occurrence of serious adverse drug reaction during the study.**
- **Worsening the disease or not responding to the treatment for at least 6 months.**

<u>ZYTUX™ NEXT COURSE ELIGIBILITY</u>

- **Patients will receive another single Rituximab infusion (1000 mg) at month 6.**
- **Repeat rituximab infusion will be administered sooner if myasthenic symptoms reappear and interfere with daily life activities of patient.**
- **The interval between Rituximab infusions should be at least three months.**

SCREENING DAY -10 to 0

PARTICIPANT ELIGIBILITY REVIEW

	End of Screening Visit Checklist	Yes	No
1	Does the participant satisfy the inclusion and exclusion criteria to date?		
2	Have all Screening Visit procedures been completed?		
3	Have the Medical History and Concomitant Medication pages been completed?		
4	Is the participant still willing to proceed in the trial?		

**Participant's eligibility Investigator Sign-Off:**
Is the participant eligible to take part in the Clinical Trial?<br>□ Yes □ No, Please give reason for screen failure below
Investigator's Signature: ___________________ Date: ------/--/-- (year/ month/day)<br><br>Investigator's Name:
Reason(s) for screen failure:
1.
2.
3.

SCREENING DAY -10 to 0

LABORATORY DATA

**BUN:**	**Sodium(serum):**
**Creatinine:**	**Potassium (serum):**
**AST:**	**ALP:**
**ALT:**	**Bilirubin:**
**CBC with differential:**<br>**WBC: ………….** /µL<br>**Hb: …………….** /dL<br>**HCT: ………….** %<br>**MCV: ………….** fL<br>**MCHC: ………..** g/dL<br>**PLT: ………….** /µL	

If other lab data has been taken due to patient`s condition/background diseases, please write down the corresponding lab results: --------------

Date (dd/mm/yy): |___|___|/|___|___|/|___|___|

SCREENING DAY -10 to 0

MONITORING PARAMETER

Subject ID:------------

Subject number:--------------

Name of lab that test were done?----------------------

Date of test:----------------

Anti-MuSK antibody:---------------------

Anti-AChR antibody:---------------------

Has thymectomy been performed on the patient?

No □ Yes □

If answer is yes, please mention the date of this operation: ………

Pathology

SCREENING DAY -10 to 0

Visit:-------

Current date:--------

Subject ID:-------------, Subject number:-------------

Enter score here:----------------------

پرسشنامه کیفیت زندگی میاستنی گراویس(MG-QOL15):

لطفا مشخص کنید هر جمله تا چه حد درست است (در چند هفته قبل).

	هرگز	کمی	تا حدودی	زیاد	بسیار زیاد
	۰	۱	۲	۳	۴
۱. من از بیماری میاستنی ام خسته شدم.					
۲. من در استفاده از چشمانم مشکل دارم.					
۳. من به علت بیماری میاستنی در غذا خوردن مشکل دارم.					
۴. من به علت بیماری میاستنی ام، فعالیتهای اجتماعی‌ام را محدود کردم.					
۵. بیماری میاستنی توانایی من را برای لذت بردن از سرگرمی ها محدود می کند.					
۶. من به علت بیماری میاستنی ام در برآورده کردن نیازهای خانواده ام مشکل دارم.					
۷. من باید برای بیماری میاستنی خود چاره ای بیاندیشم.					
۸. بیماری میاستنی بر روی مهارتهای کاری و موقعیت شغلی من تاثیر منفی داشته است.					
۹. من به علت بیماری میاستنی ام در صحبت کردن مشکل دارم.					

					۱۰. من به علت بیماری میاستنی ام در رانندگی مشکل دارم.
					۱۱. من از بابت بیماری میاستنی ام افسرده هستم.
					۱۲. من به علت بیماری میاستنی ام در راه رفتن مشکل دارم.
					۱۳. من به علت بیماری میاستنی ام برای حضور در اماکن عمومی مشکل دارم.
					۱۴. بیماری میاستنی زندگی من را تحت الشعاع قرار داده است.
					۱۵. من در انجام نظافت شخصی ام مشکل دارم.

	**نمره کل**

SCREENING DAY -10 to 0

Visit:-------

Current date:--------

Subject ID:------------- Subject number:-------------

Enter score here:----------------------

MG Activities of Daily Living Scale: (MG-ADL)

Grade	0	1	2	3	Score (0, 1, 2, 3)
**Talking**	Normal	Intermittent slurring of nasal speech	Constant slurring or nasal, but can be understood	Difficult to understand speech	
**Chewing**	Normal	Fatigue with solid food	Fatigue with soft food	Gastric tube	
**Swallowing**	Normal	Rare episode of choking	Frequent choking necessitating	Gastric tube	
**Breathing**	Normal	Shortness of breath with exertion	Shortness of breath at rest	Ventilator dependence	
**Impairment of ability to brush teeth or comb hair**	none	Extra effort, but no rest periods needed	Rest periods needed	Cannot do one of these functions	
**Impairment of ability to arise from a chair**	none	Mild, sometimes uses arms	Moderate, always uses arms	Severe, requires assistance	
**Double vision**	none	Occurs, but not daily	Occurs, but not daily	Constant	
**Eyelid droop**	None	Occurs, but not daily	Daily, but not constant	Constant	

Total score	

Date (dd/mm/yy): |___|___|/|___|___|/|___|___|

SCREENING DAY -10 to 0

Visit:-------

Current date:-------- Subject ID:------------,

Subject number:-------------

Enter score here:----------------------

**MGFA Clinical Classification**	
**Class I**	Any ocular muscle weakness<br>May have weakness of eye closure<br>Strength of all the other muscles is normal
**Class II**	Mild weakness affecting muscles other than ocular muscles<br>May also have ocular muscle weakness of any severity
**IIa**	Predominantly affecting limb, axial muscles, or both.<br>May also have lesser involvement of oropharyngeal muscles
**IIb**	Predominantly affecting oropharyngeal, respiratory muscles, or both<br>May also have lesser or equal involvement of limb, axial muscles, or both
**Class III**	Moderate weakness affecting muscles other than ocular muscles<br>May also have ocular muscle weakness of any severity
**IIIa**	Predominantly affecting limb, axial muscles, or both<br>May also have lesser involvement of oropharyngeal muscles
**IIIb**	Predominantly affecting oropharyngeal, respiratory muscles, or both<br>May also have lesser or equal involvement of limb, axial muscles, or both
**Class IV**	Severe weakness affecting muscles other than ocular muscles<br>May also have ocular muscle weakness of any severity
**IVa**	Predominantly affecting limb, axial muscles, or both<br>May also have lesser involvement of oropharyngeal muscles
**IVb**	Predominantly affecting oropharyngeal, respiratory muscles, or both<br>May also have lesser or equal involvement of limb, axial muscles, or both
**Class V**	Defined as intubation, with or without mechanical ventilation, except when employed during routine postoperative management. The use of a feeding tube without intubation places the patient in class IVb.

SCREENING DAY -10 to 0

Visit:-------

Current date:--------

Subject ID:-------------, Subject number:-------------

The Myasthenia gravis Composite scale (MG Composite)

**Ptosis, upward (physician examination)**	$> 45\ seconds = 0$	11-45 seconds = 1	1-10 seconds = 2	Immediate = 3
**Double vision on lateral gaze, left or right (physician examination)**	$> 45\ seconds = 0$	11-45 seconds = 1	1-10 seconds = 3	Immediate = 4
**Eye closure (physician examination)**	Normal = 0	Mild weakness (can be forced open with effort) = 0	Moderate weakness (can be forced open easily) = 1	Severe weakness (unable to keep eye closed) = 2
**Talking (patient history)**	Normal = 0	Intermittent slurring or nasal speech = 2	Constant slurring or nasal but can be understood = 4	Difficult to understand speech = 6
**Chewing (patient history)**	Normal = 0	Fatigue with solid food = 2	Fatigue with solid food = 4	Gastric tube = 6
**Swallowing (patient history)**	Normal = 0	Rare episode of choking or trouble = 2	Frequent trouble swallowing e.g. necessitating changes in diet = 5	Gastric tube = 6
**Breathing (thought to be caused by MG)**	Normal = 0	Shortness of breath with exertion = 2	Shortness of breath at rest = 4	Ventilator dependence = 9
**Neck flexion or extension (weakest) (physician examination)**	Normal = 0	Mild weakness = 1	Moderate weakness (i.e. $\sim 50\% weak \pm 15\%) = 3$[a]	Severe weakness = 4
**Shoulder abduction (physician examination)**	Normal = 0	Mild weakness = 2	Moderate weakness (i.e. $\sim 50\% weak \pm 15\%) = 4$[a]	Severe weakness = 5
**Hip flexion (physician examination)**	Normal = 0	Mild weakness = 2	Moderate weakness (i.e. $\sim 50\% weak \pm 15\%) = 4$[a]	Severe weakness = 5

[a] Moderate weakness for neck and limb items should be construed as weakness that equals roughly 50% _15% of expected normal strength. Any weakness milder than that would be mild and any weakness more severe than that would be classified as severe.

Total score	

SCREENING DAY -10 to 0

PRINCIPILE INVESTIGATORE'S SIGN OFF

Principal Investigator's Signature Statement:	
I have reviewed this CRF and confirm that, to the best of my knowledge, it accurately reflects the study information obtained for this participant. All entries were made either by me or by a person under my supervision who has signed the Delegation and Signature Log.	
Principal Investigator's Signature:<br><br>Principal Investigator's Name:	Date of Signature:<br>-------/----/---- (year/month/day)
**ONCE SIGNED, NO FURTHER CHANGES CAN BE MADE TO THIS CRF WITHOUT A SIGNED DATA QUERY FORM.**	

VISIT 1 (Week 1)

VITAL SIGNS	
Temperature:<br><br>Respiration rate:	Blood Pressure:<br><br>Heart rate:

PHYSICAL EXAMINATION

Examine the following and place a √ in the appropriate column. If abnormal is √, then provide the condition(s) in the comments column as provided.

Body system	Normal	Abnormal	Not done	comments
Appearance				
Skin				
HEENT				
Thyroid				
Chest				
Renal system				
Cardiovascular system				
Breasts				
Heart				
Abdomen				
Musculoskeletal				
Genitalia				
Pelvic				
Rectal				
Prostate				
Vascular				
Neurological				
Lymph nodes				
specify, others				

VISIT 1 (Week 1)

CONCOMITANT MEDICATION

Has the participant used any Concomitant Medications? **No □ Yes □,** Complete below

CM No.	Medication name (Record <specify Generic or Brand> name)	Start date (year/month/day)	Stop date (year/month/day)	Or tick if ongoing at end of study?	Reason for use (Enter related AE diagnosis, or other reasons for use, e.g. Prophylaxis)	Dose (Units)	Route	Frequency
1								
2								
3								
4								
5								
6								
7								
8								
9								
10								
11								
12								
13								
14								
15								
16								

VISIT 1 (Week 1)

RELAPSE RECORD FORM AFTER TREATMENT

Please just mention any relapse after previous visit

Relapse	Date	Ocular Myasthenia Gravis	Generalized Myasthenia Gravis	Duration of relapse	Treatment

CORTICOSTROID`S DOSAGE CHANGE

Has administrated corticosteroid's dosage been changed?

No □ Yes □

If answer is yes, please mention the new dose----------------

PYRIDOSTIGMINE`S DOSAGE CHANGE

Has administrated pyridostigmine `s dosage been changed?

No □ Yes □

If answer is yes, please mention the new dose----------------

Date (dd/mm/yy): |___|___|/|___|___|/|___|___|

VISIT 1 (Week 1)

LABORATORY DATA

**BUN:**	**Sodium(serum):**
**Creatinine:**	**Potassium (serum):**
**AST:**	**ALP:**
**ALT:**	**Bilirubin:**
**CBC with differential:**<br>**WBC: ………….** /μL<br>**Hb: …………….** /dL<br>**HCT: ………….** %<br>**MCV: ………….** fL<br>**MCHC: ………..** g/dL<br>**PLT: ………….** /μL	

If other lab data has been taken due to patient`s condition/background diseases, please write down the corresponding lab results: ---------------

Date (dd/mm/yy): |___|___|/|___|___|/|___|___|

VISIT 1 (Week 1)

Evaluation of taking IVIG and Plasmapheresis:

Has patient received IVIG from the last visit?

No □   Yes □

If yes, please mention the dose and number of administrations:

Has patient undertaken plasmapheresis from the last visit?

No □   Yes □

If yes, please mention the number of this operation:

VISIT 1 (Week 1)

RITUXIMAB INFUSION FORM

Infusion date: ------------------------------- (year/ month/ day)

Place of injection: ---

Rituximab □ Induction phase □ Maintenance phase

(Please circle) week 1 2 3 4 Month ----------------

Dose of Rituximab administered on this infusion date: --------------

Was patient`s EKG taken before, during and after infusion?

□ Yes

□ No

If yes, please mention the condition of patient-----------------

If patient take antihypertensive drugs, did he/she hold the drug 12 hrs before rituximab infusion?

□ Yes

□ No

Use of premedication to prevent infusion reactions:

1) Was antihistamine drugs administered at this rituximab infusion?

No□

Yes□, please mention the name and dose: -------------------------

2) Was acetaminophen administered at this rituximab infusion?

No□

Yes□, please mention the dose: -------------------------

3) Was glucocorticoid drugs administered at this rituximab infusion?

No□

Yes□, please mention the name and dose: -------------------------

4) Was NSAIDs drugs administered at this rituximab infusion?

No□

Yes□, please mention the name and dose: -------------------------

VISIT 1 (Week 1)

Rituximab infusion speed protocol:

Total time: approximately 3 hrs

First 30 minute: 50 mg/hour (for example for 500 mg vial at 0.5 liter serum, it will be 25 ml at 30 minute that it equals 20 drops per min)

Then increased by 50 mg/hour every 30 minutes to a maximum of 400 mg/hour

Total dose: 1g IV

Was the rituximab infusion administered completely?

□Yes □No

If No, please indicate:

Nurse name and signature:

VISIT 1 (Week 1)

RITUXIMAB INFUSION REACTION

Infusion Reaction:

No □ Yes □

If answer is yes, please complete details:

Time of reaction start:

□ Within 30 min

□ Within 30min-2hrs

□ After 2 hrs

□ After 24 hrs

Signs and symptoms of infusion reactions:

□ Fever and/or shaking chills
□ Flushing and/or itching
□ Alterations in heart rate and blood pressure
□ Dyspnea or chest discomfort
□ Back or abdominal pain
□ Nausea, vomiting, and/or diarrhea
□ Various types of skin rashes
□ Others: --------

Classification of infusion reactions:

□ **Grade 1.** Mild transient reaction; infusion interruption not indicated; intervention not indicated

□ **Grade 2.** Therapy or infusion interruption indicated but responds promptly to symptomatic treatment (eg, antihistamines, NSAIDs, narcotics, intravenous fluids); prophylactic medication indicated for less than or equal to 24 hours

□ **Grade 3.** Prolonged (ie, not rapidly responsive to symptomatic medication and/or brief interruption of infusion); recurrence of symptoms following initial improvement; hospitalization indicated for other clinical sequelae

□ **Grade 4.** Life-threatening consequences; urgent intervention indicated

□ **Grade 5.** Death

Is anaphylactic reaction happened?

Yes □ No □

Repeated premedication after or during infusion reaction:

Yes □ No □

Treatment and dosage:

Does infusion temporarily interrupted and then continued?-----------------

Does infusion rate reduced and completed (mention the speed)?----------------

Does infusion rate reduced and not completed?---------------

VISIT 1 (Week 1)

SERIOUS ADVERSE EVENTS Page 1 of 2

Event Start Date: --------/----/---- (year/month/day), Event end Date: --------/----/---- (year/month/day)

Date Reported: --------/----/---- (year/month/day), Reported to staff by: ---------------------------------

Death Date (If applicable): --------/----/---- (year/month/day)

Death Occurred (check one):

□ Within 24 hours of investigational therapy □ Within 30 days of investigational therapy

□ Within 7 days of investigational therapy □ After 30 days of investigational therapy

Did the SAE occur at your site or at a site for which the PI is responsible? □ Yes □No

SAE Description/Narrative:

Treating Physician Comments:

PI Comments:

Outcome: (check one)

□ Fatal/Died
□ Intervention for AE Continuing
□ Not Recovered/Not Resolved
□ Recovered/Resolved with Sequelae
□ Recovered/Resolved without Sequelae
□ Recovering/Resolving

Consent Form Change Required? □ Yes □ No

SAE Classification: (check all that apply)

□ Fatal (resulted in death)
□ A life-threatening occurrence
□ Requires inpatient hospitalization or prolongation of existing hospitalization
□ Results in persistent or significant disability/incapacity
□ Results in congenital anomaly/birth defect
□ A significant medical incident that, based upon appropriate medical judgment, may jeopardize the subject and require medical or surgical intervention to prevent one of the outcomes listed above.
□ Loss of confidentiality that results in criminal or civil liability for participation or damage to financial standing, employability, insurability or reputation of the participant

VISIT 1 (Week 1)

SERIOUS ADVERSE EVENTS Page 2 of 2

SAE Reported Symptom:

Category: [refer to the Safety Profiler website to search the Category and Toxicity of the SAE symptom reported: http://safetyprofiler-ctep.nci.nih.gov/CTC/CTC.aspx]

Toxicity:

Grade/Severity (check one):
□ 1 - Mild
□ 2 - Moderate
□ 3 - Severe
□ 4 - Life Threatening
□ 5 – Death (Fatal)

Unexpected? □ Yes □No

Dose Limiting Toxicity (DLT)? □ Yes □ No □ Not Applicable

Action taken:

□ Dose reduced

□ Dose interrupted, then reduced

□ None

□ Regimen interrupted

□ Therapy discontinued

Primary attribution: (check one)

□ Definite

□ Probable

□ Possible

□ Unlikely

□ Unrelated

Detailed Attribution: (check one)

□ **Disease/Condition** Specify:

□ **Investigational Treatment** Specify:

□ **Non-investigational Treatment** Specify:

□ **Other** Specify:

VISIT 1 (Week 1)

ADVERSE EVENT CHECK LIST

Head and Lymphatic System	Yes	No
Lymphopenia		
Leukopenia		
Neutropenia		
Thrombocytopenia		
Anemia		

Musculoskeletal	Yes	No
Myalgia		
Arthralgia		

	Yes	No
Infusion reaction		

Nervous System	Yes	No
Dizziness		
Anxiety		
Headache		
Progressive multifocal leukoencephalopathy		

Digestive System	Yes	No
Nausea		
Diarrhea		
Vomiting		

Cardiovascular System	Yes	No
Hypotension		
Hypertension		

Skin and Appendages	Yes	No
Night sweats		
Rash		
Pruritus		
Urticaria		

Body as a Whole	Yes	No
Fever		
Chills		
Infection		
Asthenia		
Abdominal Pain		
Pain		
Back Pain		
Throat Irritation		

Metabolic and Nutritional Disorder	Yes	No
Angioedema		
Hyperglycemia		
Peripheral Edema		

VISIT 1 (Week 1)

ADVERSE EVENTS

	**Event Name** (Please give Diagnosis if known)	**Start date** (year/month/day)	**Stop date** (year/month/day)	**Serious?** If serious, please complete a JRO SAE form	**Con-comitant Medication given**	**Severity** 0 - Mild 1- Mode-rate 2 - Severe	**Study Drug Action** 0 - None 1 - Temporarily Interrupted 2 - permanently withdrawn	**Outcome** 0 - Resolved 1- Resolved with sequelea 2 - Not resolved	**Relationship to Study Drug** 0 - Definitely 1 - Probably 2 - Possibly 3 - Unlikely 4 - Not related 5 - Not assessable
1.				□ No □ Yes	□ No □ Yes				
2.				□ No □ Yes	□ No □ Yes				
3.				□ No □ Yes	□ No □ Yes				
4.				□ No □ Yes	□ No □ Yes				
5.				□ No □ Yes	□ No □ Yes				
6.				□ No □ Yes	□ No □ Yes				
7.				□ No □ Yes	□ No □ Yes				
8.				□ No □ Yes	□ No □ Yes				
9.				□ No □ Yes	□ No □ Yes				

10.				□ No<br>□ Yes	□ No<br>□ Yes				
11.				□ No<br>□ Yes	□ No<br>□ Yes				
12.				□ No<br>□ Yes	□ No<br>□ Yes				
13.				□ No<br>□ Yes	□ No<br>□ Yes				
14.				□ No<br>□ Yes	□ No<br>□ Yes				
15.				□ No<br>□ Yes	□ No<br>□ Yes				
16.				□ No<br>□ Yes	□ No<br>□ Yes				
17.				□ No<br>□ Yes	□ No<br>□ Yes				
18.				□ No<br>□ Yes	□ No<br>□ Yes				

I have reviewed the AEs on this page and have assessed them for seriousness, causality, severity and outcome and confirm that, to the best of my knowledge, it accurately reflects the study information obtained for this participant

PI signature ________________________________ _________________ Date: _________________________ Please check box if this is the last page used

VISIT 1 (Week 1)

SUBJECT OFF STUDY

Date subject went Off Study: ----------/----/----- (year/month/day)

INDICATE OFF STUDY REASON:

□ Study Activities Completed
□ Side effects of study intervention (complete applicable SAE form or AE Tracking Log)
□ Death
□ Subject lost to follow-up (provide comments below)
□ Subject refused follow-up (provide comments below)
□ Other (provide comments below)
□ Subject withdrew (complete Early Withdrawal section below)

EARLY WITHDRAWAL

Last Visit Completed: □ Early *Withdrawal form not completed*

Indicate the **primary** reason the subject has withdrawn from the study (select only one):
□ Subject deemed eligible but declined participation
□ Subject deemed inappropriate for study participation by the PI
□ Participant was determined to be ineligible after enrollment (provide comments below)
□ Identification of disease/condition after enrollment that warrants withdrawal
□ Unable to continue due to personal constraints
□ Side effects of study intervention
□ Other -----------------------

VISIT 1 (Week 1)

STANDARD VISIT SHEET

Visit Checklist:			
		Yes	No
1.	**Have there been any new Adverse Events?** (If yes, please record in Adverse Events page)		
2.	**Have there been any changes in Concomitant Medications?** (If yes, please record in Concomitant Medications Log)		
3.	**Laboratory data**		
4.	**Rituximab Administration**		
5.	**Is the patient experiencing a relapse?** **(If yes record in complete physical exam form)**		
6.	**Is Adverse event check list completed?**		
7.	**Does patient withdraw or exclude from study?**		

Date (dd/mm/yy): |___|___|/|___|___|/|___|___|

VISIT 2 (Week 3)

VITAL SIGNS	
Temperature:<br><br>Respiration rate:	Blood Pressure:<br><br>Heart rate:

PHYSICAL EXAMINATION

Examine the following and place a √ in the appropriate column. If abnormal is √, then provide the condition(s) in the comments column as provided.

Body system	Normal	Abnormal	Not done	comments
Appearance				
Skin				
HEENT				
Thyroid				
Chest				
Renal system				
Cardiovascular system				
Breasts				
Heart				
Abdomen				
Musculoskeletal				
Genitalia				
Pelvic				
Rectal				
Prostate				
Vascular				
Neurological				
Lymph nodes				
specify, others				

VISIT 2 (Week 3)

CONCOMITANT MEDICATION

Has the participant used any Concomitant Medications? **No □ Yes □**, Complete below

CM No.	Medication name (Record <specify Generic or Brand> name)	Start date (year/month/day)	Stop date (year/month/day)	Or tick if ongoing at end of study?	Reason for use (Enter related AE diagnosis, or other reasons for use, e.g. Prophylaxis)	Dose (Units)	Route	Frequency
1								
2								
3								
4								
5								
6								
7								
8								
9								
10								
11								
12								
13								
14								
15								
16								

VISIT 2 (Week 3)

RELAPSE RECORD FORM AFTER TREATMENT

Please just mention any relapse after previous visit

Relapse	Date	Ocular Myasthenia Gravis	Generalized Myasthenia Gravis	Duration of relapse	Treatment

CORTICOSTROID`S DOSAGE CHANGE

Has administrated corticosteroid's dosage been changed?

No □ Yes □

If answer is yes, please mention the new dose----------------

PYRIDOSTIGMINE`S DOSAGE CHANGE

Has administrated pyridostigmine `s dosage been changed?

No □ Yes □

If answer is yes, please mention the new dose----------------

Date (dd/mm/yy): |___|___|/|___|___|/|___|___|

VISIT 2 (Week 3)

LABORATORY DATA

**BUN:**	**Sodium(serum):**
**Creatinine:**	**Potassium (serum):**
**AST:**	**ALP:**
**ALT:**	**Bilirubin:**
**CBC with differential:**<br>**WBC: ………….** /μL<br>**Hb: …………….** /dL<br>**HCT: ………….** %<br>**MCV: ………….** fL<br>**MCHC: ………..** g/dL<br>**PLT: ………….** /μL	

If other lab data has been taken due to patient`s condition/background diseases, please write down the corresponding lab results: --------------

VISIT 2 (Week 3)

Evaluation of taking IVIG and Plasmapheresis:

Has patient received IVIG from the last visit?

No □ Yes □

If yes, please mention the dose and number of administrations:

Has patient undertaken plasmapheresis from the last visit?

No □ Yes □

If yes, please mention the number of this operation:

VISIT 2 (Week 3)

RITUXIMAB INFUSION FORM

Infusion date: ------------------------------- (year/ month/ day)

Place of injection: ---

Rituximab □ Induction phase **□ Maintenance phase**

(Please circle) week 1 2 3 4 **Month ----------------**

Dose of Rituximab administered on this infusion date: --------------

Was patient`s EKG taken before, during and after infusion?

□ Yes

□ No

If yes, please mention the condition of patient-----------------

If patient take antihypertensive drugs, did he/she hold the drug 12 hrs before rituximab infusion?

□ Yes

□ No

Use of premedication to prevent infusion reactions:

1) Was antihistamine drugs administered at this rituximab infusion?

No□

Yes□, please mention the name and dose: -------------------------

2) Was acetaminophen administered at this rituximab infusion?

No□

Yes□, please mention the dose: -------------------------

3) Was glucocorticoid drugs administered at this rituximab infusion?

No□

Yes□, please mention the name and dose: -------------------------

4) Was NSAIDs drugs administered at this rituximab infusion?

No□

Yes□, please mention the name and dose: -------------------------

VISIT 2 (Week 3)

Rituximab infusion speed protocol:

Total time: approximately 3 hrs

First 30 minute: 50 mg/hour (for example for 500 mg vial at 0.5 liter serum, it will be 25 ml at 30 minute that it equals 20 drops per min)

Then increased by 50 mg/hour every 30 minutes to a maximum of 400 mg/hour

Was the rituximab infusion administered completely?

□Yes □No

If No, please indicate:

Nurse name and signature:

VISIT 2 (Week 3)

RITUXIMAB INFUSION REACTION

Infusion Reaction:

No □ Yes □

If answer is yes, please complete details:

Time of reaction start:

□ Within 30 min

□ Within 30min-2hrs

□ After 2 hrs

□ After 24 hrs

Signs and symptoms of infusion reactions:

- □ Fever and/or shaking chills
- □ Flushing and/or itching
- □ Alterations in heart rate and blood pressure
- □ Dyspnea or chest discomfort
- □ Back or abdominal pain
- □ Nausea, vomiting, and/or diarrhea
- □ Various types of skin rashes
- □ Others:--------

Classification of infusion reactions:

□ **Grade 1.** Mild transient reaction; infusion interruption not indicated; intervention not indicated

□ **Grade 2.** Therapy or infusion interruption indicated but responds promptly to symptomatic treatment (eg, antihistamines, NSAIDs, narcotics, intravenous fluids); prophylactic medication indicated for less than or equal to 24 hours

□ **Grade 3.** Prolonged (ie, not rapidly responsive to symptomatic medication and/or brief interruption of infusion); recurrence of symptoms following initial improvement; hospitalization indicated for other clinical sequelae

□ **Grade 4.** Life-threatening consequences; urgent intervention indicated

□ **Grade 5.** Death

Is anaphylactic reaction happened?

Yes □ No □

Repeated premedication after or during infusion reaction:

Yes □ No □

Treatment and dosage:

Does infusion temporarily interrupted and then continued?-----------------

Does infusion rate reduced and completed (mention the speed)?----------------

Does infusion rate reduced and not completed?---------------

VISIT 2 (Week 3)

SERIOUS ADVERSE EVENTS Page 1 of 2

Event Start Date: --------/----/---- (year/month/day), Event end Date: --------/----/---- (year/month/day)

Date Reported: --------/----/---- (year/month/day), Reported to staff by: ---------------------------------

Death Date (If applicable): --------/----/---- (year/month/day)

Death Occurred (check one):

□ Within 24 hours of investigational therapy □ Within 30 days of investigational therapy

□ Within 7 days of investigational therapy □ After 30 days of investigational therapy

Did the SAE occur at your site or at a site for which the PI is responsible? □Yes □No

SAE Description/Narrative:

Treating Physician Comments:

PI Comments:

Outcome: (check one)

□ Fatal/Died
□ Intervention for AE Continuing
□ Not Recovered/Not Resolved
□ Recovered/Resolved with Sequelae
□ Recovered/Resolved without Sequelae
□ Recovering/Resolving

Consent Form Change Required? □ Yes □ No

SAE Classification: (check all that apply)

□ Fatal (resulted in death)
□ A life-threatening occurrence
□ Requires inpatient hospitalization or prolongation of existing hospitalization
□ Results in persistent or significant disability/incapacity
□ Results in congenital anomaly/birth defect
□ A significant medical incident that, based upon appropriate medical judgment, may jeopardize the subject and require medical or surgical intervention to prevent one of the outcomes listed above.
□ Loss of confidentiality that results in criminal or civil liability for participation or damage to financial standing, employability, insurability or reputation of the participant

VISIT 2 (Week 3)

SERIOUS ADVERSE EVENTS Page 2 of 2

SAE Reported Symptom:

Category: [refer to the Safety Profiler website to search the Category and Toxicity of the SAE symptom reported: http://safetyprofiler-ctep.nci.nih.gov/CTC/CTC.aspx]

Toxicity:

Grade/Severity (check one):
□ 1 - Mild
□ 2 - Moderate
□ 3 - Severe
□ 4 - Life Threatening
□ 5 – Death (Fatal)

Unexpected? □ Yes □No

Dose Limiting Toxicity (DLT)? □ Yes □ No □ Not Applicable

Action taken:

□ Dose reduced

□ Dose interrupted, then reduced

□ None

□ Regimen interrupted

□ Therapy discontinued

Primary attribution: (check one)

□ Definite

□ Probable

□ Possible

□ Unlikely

□ Unrelated

Detailed Attribution: (check one)

□ **Disease/Condition** Specify:

□ **Investigational Treatment** Specify:

□ **Non-investigational Treatment** Specify:

□ **Other** Specify:

VISIT 2 (Week 3)

ADVERSE EVENT CHECK LIST

Head and Lymphatic System	Yes	No
Lymphopenia		
Leukopenia		
Neutropenia		
Thrombocytopenia		
Anemia		

Digestive System	Yes	No
Nausea		
Diarrhea		
Vomiting		

Skin and Appendages	Yes	No
Night sweats		
Rash		
Pruritus		
Urticaria		

Metabolic and Nutritional Disorder	Yes	No
Angioedema		
Hyperglycemia		
Peripheral Edema		

Musculoskeletal	Yes	No
Myalgia		
Arthralgia		

	Yes	No
Infusion reaction		

Nervous System	Yes	No
Dizziness		
Anxiety		
Headache		
Progressive multifocal leukoencephalopathy		

Cardiovascular System	Yes	No
Hypotension		
Hypertension		

Body as a Whole	Yes	No
Fever		
Chills		
Infection		
Asthenia		
Abdominal Pain		
Pain		
Back Pain		
Throat Irritation		

Patient name/Surname: ………………. Study Site: ……………….

VISIT 2 (Week 3)

ADVERSE EVENTS

	**Event Name** (Please give Diagnosis if known)	**Start date** (year/month/day)	**Stop date** (year/month/day)	**Serious?** If serious, please complete a JRO SAE form	**Con-comitant Medication given**	**Severity** 0 - Mild 1- Mode-rate 2 - Severe	**Study Drug Action** 0 - None 1 - Temporarily Interrupted 2 - permanently withdrawn	**Outcome** 0 - Resolved 1- Resolved with sequelea 2 - Not resolved	**Relationship to Study Drug** 0 - Definitely 1 - Probably 2 - Possibly 3 - Unlikely 4 - Not related 5 - Not assessable
1.				□ No □ Yes	□ No □ Yes				
2.				□ No □ Yes	□ No □ Yes				
3.				□ No □ Yes	□ No □ Yes				
4.				□ No □ Yes	□ No □ Yes				
5.				□ No □ Yes	□ No □ Yes				
6.				□ No □ Yes	□ No □ Yes				
7.				□ No □ Yes	□ No □ Yes				
8.				□ No □ Yes	□ No □ Yes				
9.				□ No □ Yes	□ No □ Yes				

Date (dd/mm/yy): |___|___|/|___|___|/|___|___|

10.				□ No<br>□ Yes	□ No<br>□ Yes				
11.				□ No<br>□ Yes	□ No<br>□ Yes				
12.				□ No<br>□ Yes	□ No<br>□ Yes				
13.				□ No<br>□ Yes	□ No<br>□ Yes				
14.				□ No<br>□ Yes	□ No<br>□ Yes				
15.				□ No<br>□ Yes	□ No<br>□ Yes				
16.				□ No<br>□ Yes	□ No<br>□ Yes				
17.				□ No<br>□ Yes	□ No<br>□ Yes				
18.				□ No<br>□ Yes	□ No<br>□ Yes				

I have reviewed the AEs on this page and have assessed them for seriousness, causality, severity and outcome and confirm that, to the best of my knowledge, it accurately reflects the study information obtained for this participant

PI signature ________________________________ _________________ Date: __________________________ Please check box if this is the last page used

VISIT 2 (Week 3)

SUBJECT OFF STUDY

Date subject went Off Study: ----------/----/----- (year/month/day)

INDICATE OFF STUDY REASON:

□ Study Activities Completed
□ Side effects of study intervention (complete applicable SAE form or AE Tracking Log)
□ Death
□ Subject lost to follow-up (provide comments below)
□ Subject refused follow-up (provide comments below)
□ Other (provide comments below)
□ Subject withdrew (complete Early Withdrawal section below)

EARLY WITHDRAWAL

Last Visit Completed: □ Early *Withdrawal form not completed*

Indicate the **primary** reason the subject has withdrawn from the study (select only one):
□ Subject deemed eligible but declined participation
□ Subject deemed inappropriate for study participation by the PI
□ Participant was determined to be ineligible after enrollment (provide comments below)
□ Identification of disease/condition after enrollment that warrants withdrawal
□ Unable to continue due to personal constraints
□ Side effects of study intervention
□ Other -----------------------

VISIT 2 (Week 3)

STANDARD VISIT SHEET

Visit Checklist:			
		Yes	No
1.	**Have there been any new Adverse Events?**<br>(If yes, please record in Adverse Events page)		
2.	**Have there been any changes in Concomitant Medications?**<br>(If yes, please record in Concomitant Medications Log)		
3.	**Laboratory data**		
4.	**Rituximab Administration**		
5.	**Is the patient experiencing a relapse?**<br>**(If yes record in complete physical exam form)**		
6.	**Is Adverse event check list completed?**		
7.	**Does patient withdraw or exclude from study?**		

Date (dd/mm/yy): |___|___|/|___|___|/|___|___|

VISIT 3 (Week 7)

MONITORING PARAMETER

Subject ID:-------------

Subject number:---------------

Name of lab that test were done?----------------------

Date of test:-----------------

CD 19:-------------------------

CD 20:-------------------------

VISIT 4 (Week 12)

VITAL SIGNS	
Temperature:<br><br>Respiration rate:	Blood Pressure:<br><br>Heart rate:

PHYSICAL EXAMINATION

Examine the following and place a √ in the appropriate column. If abnormal is √, then provide the condition(s) in the comments column as provided.

Body system	Normal	Abnormal	Not done	comments
Appearance				
Skin				
HEENT				
Thyroid				
Chest				
Renal system				
Cardiovascular system				
Breasts				
Heart				
Abdomen				
Musculoskeletal				
Genitalia				
Pelvic				
Rectal				
Prostate				
Vascular				
Neurological				
Lymph nodes				
specify, others				

VISIT 4 (Week 12)

CONCOMITANT MEDICATION

Has the participant used any Concomitant Medications? **No □ Yes □,** Complete below

CM No.	Medication name (Record <specify Generic or Brand> name)	Start date (year/month/day)	Stop date (year/month/day)	Or tick if ongoing at end of study?	Reason for use (Enter related AE diagnosis, or other reasons for use, e.g. Prophylaxis)	Dose (Units)	Route	Frequency
1								
2								
3								
4								
5								
6								
7								
8								
9								
10								
11								
12								
13								
14								
15								
16								

Date (dd/mm/yy): |___|___|/|___|___|/|___|___|

Patient name/Surname: ………………. Study Site: ……………….

VISIT 4 (Week 12)

RELAPSE RECORD FORM AFTER TREATMENT

Please just mention any relapse after previous visit

Relapse	Date	Ocular Myasthenia Gravis	Generalized Myasthenia Gravis	Duration of relapse	Treatment

CORTICOSTROID`S DOSAGE CHANGE

Has administrated corticosteroid's dosage been changed?

No □ Yes □

If answer is yes, please mention the new dose----------------

PYRIDOSTIGMINE`S DOSAGE CHANGE

Has administrated pyridostigmine `s dosage been changed?

No □ Yes □

If answer is yes, please mention the new dose----------------

Date (dd/mm/yy): |___|___|/|___|___|/|___|___|

VISIT 4 (Week 12)

LABORATORY DATA

**BUN:**	**Sodium(serum):**
**Creatinine:**	**Potassium (serum):**
**AST:**	**ALP:**
**ALT:**	**Bilirubin:**
**CBC with differential:**<br>**WBC: ………….** /μL<br>**Hb: …………….** /dL<br>**HCT: ………….** %<br>**MCV: ………….** fL<br>**MCHC: ………..** g/dL<br>**PLT: ………….** /μL	

If other lab data has been taken due to patient`s condition/background diseases, please write down the corresponding lab results: ---------------

VISIT 4 (Week 12)

Evaluation of taking IVIG and Plasmapheresis:

Has patient received IVIG from the last visit?

No □ Yes □

If yes, please mention the dose and number of administrations:

Has patient undertaken plasmapheresis from the last visit?

No □ Yes □

If yes, please mention the number of this operation:

VISIT 4 (Week 12)

Visit:-------

Current date:--------

Subject ID:-------------,   Subject number:-------------

Enter score here:----------------------

پرسشنامه کیفیت زندگی میاستنی گراویس(MG-QOL15):

لطفا مشخص کنید هر جمله تا چه حد درست است (در چند هفته قبل).

	هرگز	کمی	تا حدودی	زیاد	بسیار زیاد
	۰	۱	۲	۳	۴
۱. من از بیماری میاستنی ام خسته شدم.					
۲. من در استفاده از چشمانم مشکل دارم.					
۳. من به علت بیماری میاستنی در غذا خوردن مشکل دارم.					
۴. من به علت بیماری میاستنی ام، فعالیتهای اجتماعی ام را محدود کردم.					
۵. بیماری میاستنی توانایی من را برای لذت بردن از سرگرمی ها محدود می کند.					
۶. من به علت بیماری میاستنی ام در برآورده کردن نیازهای خانواده ام مشکل دارم.					
۷. من باید برای بیماری میاستنی خود چاره ای بیاندیشم.					
۸. بیماری میاستنی بر روی مهارتهای کاری و موقعیت شغلی من تاثیر منفی داشته است.					
۹. من به علت بیماری میاستنی ام در صحبت کردن مشکل دارم.					

۱۰. من به علت بیماری میاستنی ام در رانندگی مشکل دارم.					
۱۱. من از بابت بیماری میاستنی ام افسرده هستم.					
۱۲. من به علت بیماری میاستنی ام در راه رفتن مشکل دارم.					
۱۳. من به علت بیماری میاستنی ام برای حضور در اماکن عمومی مشکل دارم.					
۱۴. بیماری میاستنی زندگی من را تحت الشعاع قرار داده است.					
۱۵. من در انجام نظافت شخصی ام مشکل دارم.					

**نمره کل**	

VISIT 4 (Week 12)

Visit:-------

Current date:--------

Subject ID:-------------, Subject number:--------------

Enter score here:-----------------------

MG Activities of Daily Living Scale: (MG-ADL)

Grade	0	1	2	3	Score (0, 1, 2, 3)
**Talking**	Normal	Intermittent slurring of nasal speech	Constant slurring or nasal, but can be understood	Difficult to understand speech	
**Chewing**	Normal	Fatigue with solid food	Fatigue with soft food	Gastric tube	
**Swallowing**	Normal	Rare episode of choking	Frequent choking necessitating	Gastric tube	
**Breathing**	Normal	Shortness of breath with exertion	Shortness of breath at rest	Ventilator dependence	
**Impairment of ability to brush teeth or comb hair**	none	Extra effort, but no rest periods needed	Rest periods needed	Cannot do one of these functions	
**Impairment of ability to arise from a chair**	none	Mild, sometimes uses arms	Moderate, always uses arms	Severe, requires assistance	
**Double vision**	none	Occurs, but not daily	Occurs, but not daily	Constant	
**Eyelid droop**	None	Occurs, but not daily	Daily, but not constant	Constant	

Total score	

VISIT 4 (Week 12)

Visit:-------

Current date:-------- Subject ID:------------,

Subject number:-------------

Enter score here:----------------------

**MGFA Clinical Classification**	
**Class I**	Any ocular muscle weakness<br>May have weakness of eye closure<br>Strength of all the other muscles is normal
**Class II**	Mild weakness affecting muscles other than ocular muscles<br>May also have ocular muscle weakness of any severity
**IIa**	Predominantly affecting limb, axial muscles, or both.<br>May also have lesser involvement of oropharyngeal muscles
**IIb**	Predominantly affecting oropharyngeal, respiratory muscles, or both<br>May also have lesser or equal involvement of limb, axial muscles, or both
**Class III**	Moderate weakness affecting muscles other than ocular muscles<br>May also have ocular muscle weakness of any severity
**IIIa**	Predominantly affecting limb, axial muscles, or both<br>May also have lesser involvement of oropharyngeal muscles
**IIIb**	Predominantly affecting oropharyngeal, respiratory muscles, or both<br>May also have lesser or equal involvement of limb, axial muscles, or both
**Class IV**	Severe weakness affecting muscles other than ocular muscles<br>May also have ocular muscle weakness of any severity
**IVa**	Predominantly affecting limb, axial muscles, or both<br>May also have lesser involvement of oropharyngeal muscles
**IVb**	Predominantly affecting oropharyngeal, respiratory muscles, or both<br>May also have lesser or equal involvement of limb, axial muscles, or both
**Class V**	Defined as intubation, with or without mechanical ventilation, except when employed during routine postoperative management. The use of a feeding tube without intubation places the patient in class IVb.

VISIT 4 (Week 12)

Visit:-------

Current date:--------

Subject ID:-------------, Subject number:--------------

The Myasthenia gravis Composite scale (MG Composite)

**Ptosis, upward (physician examination)**	$> 45\ seconds = 0$	11-45 seconds = 1	1-10 seconds = 2	Immediate = 3
**Double vision on lateral gaze, left or right (physician examination)**	$> 45\ seconds = 0$	11-45 seconds = 1	1-10 seconds = 3	Immediate = 4
**Eye closure (physician examination)**	Normal = 0	Mild weakness (can be forced open with effort) = 0	Moderate weakness (can be forced open easily) = 1	Severe weakness (unable to keep eye closed) = 2
**Talking (patient history)**	Normal = 0	Intermittent slurring or nasal speech = 2	Constant slurring or nasal but can be understood = 4	Difficult to understand speech = 6
**Chewing (patient history)**	Normal = 0	Fatigue with solid food = 2	Fatigue with solid food = 4	Gastric tube = 6
**Swallowing (patient history)**	Normal = 0	Rare episode of choking or trouble = 2	Frequent trouble swallowing e.g. necessitating changes in diet = 5	Gastric tube = 6
**Breathing (thought to be caused by MG)**	Normal = 0	Shortness of breath with exertion = 2	Shortness of breath at rest = 4	Ventilator dependence = 9
**Neck flexion or extension (weakest) (physician examination)**	Normal = 0	Mild weakness = 1	Moderate weakness (i.e. $\sim 50\% weak \pm 15\%) = 3$[a]	Severe weakness = 4
**Shoulder abduction (physician examination)**	Normal = 0	Mild weakness = 2	Moderate weakness (i.e. $\sim 50\% weak \pm 15\%) = 4$[a]	Severe weakness = 5
**Hip flexion (physician examination)**	Normal = 0	Mild weakness = 2	Moderate weakness (i.e. $\sim 50\% weak \pm 15\%) = 4$[a]	Severe weakness = 5

[a] Moderate weakness for neck and limb items should be construed as weakness that equals roughly 50% _15% of expected normal strength. Any weakness milder than that would be mild and any weakness more severe than that would be classified as severe.

Total score	

VISIT 4 (Week 12)

SERIOUS ADVERSE EVENTS Page 1 of 2

Event Start Date: --------/----/---- (year/month/day), Event end Date: --------/----/---- (year/month/day)

Date Reported: --------/----/---- (year/month/day), Reported to staff by: ---------------------------------

Death Date (If applicable): --------/----/---- (year/month/day)

Death Occurred (check one):

□ Within 24 hours of investigational therapy □ Within 30 days of investigational therapy

□ Within 7 days of investigational therapy □ After 30 days of investigational therapy

Did the SAE occur at your site or at a site for which the PI is responsible? □ Yes □No

SAE Description/Narrative:

Treating Physician Comments:

PI Comments:

Outcome: (check one)

□ Fatal/Died
□ Intervention for AE Continuing
□ Not Recovered/Not Resolved
□ Recovered/Resolved with Sequelae
□ Recovered/Resolved without Sequelae
□ Recovering/Resolving

Consent Form Change Required? □ Yes □ No

SAE Classification: (check all that apply)

□ Fatal (resulted in death)
□ A life-threatening occurrence
□ Requires inpatient hospitalization or prolongation of existing hospitalization
□ Results in persistent or significant disability/incapacity
□ Results in congenital anomaly/birth defect
□ A significant medical incident that, based upon appropriate medical judgment, may jeopardize the subject and require medical or surgical intervention to prevent one of the outcomes listed above.
□ Loss of confidentiality that results in criminal or civil liability for participation or damage to financial standing, employability, insurability or reputation of the participant

VISIT 4 (Week 12)

SERIOUS ADVERSE EVENTS Page 2 of 2

SAE Reported Symptom:

Category: [refer to the Safety Profiler website to search the Category and Toxicity of the SAE symptom reported: http://safetyprofiler-ctep.nci.nih.gov/CTC/CTC.aspx]

Toxicity:

Grade/Severity (check one):
□ 1 - Mild
□ 2 - Moderate
□ 3 - Severe
□ 4 - Life Threatening
□ 5 – Death (Fatal)

Unexpected? □ Yes □No

Dose Limiting Toxicity (DLT)? □ Yes □ No □ Not Applicable

Action taken:

□ Dose reduced

□ Dose interrupted, then reduced

□ None

□ Regimen interrupted

□ Therapy discontinued

Primary attribution: (check one)

□ Definite

□ Probable

□ Possible

□ Unlikely

□ Unrelated

Detailed Attribution: (check one)

□ **Disease/Condition** Specify:

□ **Investigational Treatment** Specify:

□ **Non-investigational Treatment** Specify:

□ **Other** Specify:

Date (dd/mm/yy): |___|___|/|___|___|/|___|___|

VISIT 4 (Week 12)

ADVERSE EVENT CHECK LIST

Head and Lymphatic System	Yes	No
Lymphopenia		
Leukopenia		
Neutropenia		
Thrombocytopenia		
Anemia		

Musculoskeletal	Yes	No
Myalgia		
Arthralgia		

	Yes	No
Infusion reaction		

Nervous System	Yes	No
Dizziness		
Anxiety		
Headache		
Progressive multifocal leukoencephalopathy		

Digestive System	Yes	No
Nausea		
Diarrhea		
Vomiting		

Cardiovascular System	Yes	No
Hypotension		
Hypertension		

Skin and Appendages	Yes	No
Night sweats		
Rash		
Pruritus		
Urticaria		

Body as a Whole	Yes	No
Fever		
Chills		
Infection		
Asthenia		
Abdominal Pain		
Pain		
Back Pain		
Throat Irritation		

Metabolic and Nutritional Disorder	Yes	No
Angioedema		
Hyperglycemia		
Peripheral Edema		

VISIT 4 (Week 12)

ADVERSE EVENTS

	**Event Name** (Please give Diagnosis if known)	**Start date** (year/month/day)	**Stop date** (year/month/day)	**Serious?** If serious, please complete a JRO SAE form	**Con-comitant Medication given**	**Severity** 0 - Mild 1- Mode-rate 2 - Severe	**Study Drug Action** 0 - None 1 - Temporarily Interrupted 2 - permanently withdrawn	**Outcome** 0 - Resolved 1- Resolved with sequelea 2 - Not resolved	**Relationship to Study Drug** 0 - Definitely 1 - Probably 2 - Possibly 3 - Unlikely 4 - Not related 5 - Not assessable
1.				□ No □ Yes	□ No □ Yes				
2.				□ No □ Yes	□ No □ Yes				
3.				□ No □ Yes	□ No □ Yes				
4.				□ No □ Yes	□ No □ Yes				
5.				□ No □ Yes	□ No □ Yes				
6.				□ No □ Yes	□ No □ Yes				
7.				□ No □ Yes	□ No □ Yes				
8.				□ No □ Yes	□ No □ Yes				
9.				□ No □ Yes	□ No □ Yes				

Date (dd/mm/yy): |___|___|/|___|___|/|___|___|

10.				□ No<br>□ Yes	□ No<br>□ Yes				
11.				□ No<br>□ Yes	□ No<br>□ Yes				
12.				□ No<br>□ Yes	□ No<br>□ Yes				
13.				□ No<br>□ Yes	□ No<br>□ Yes				
14.				□ No<br>□ Yes	□ No<br>□ Yes				
15.				□ No<br>□ Yes	□ No<br>□ Yes				
16.				□ No<br>□ Yes	□ No<br>□ Yes				
17.				□ No<br>□ Yes	□ No<br>□ Yes				
18.				□ No<br>□ Yes	□ No<br>□ Yes				

I have reviewed the AEs on this page and have assessed them for seriousness, causality, severity and outcome and confirm that, to the best of my knowledge, it accurately reflects the study information obtained for this participant

PI signature ________________________________ _________________ Date:_________________________ Please check box if this is the last page used

VISIT 4 (Week 12)

SUBJECT OFF STUDY

Date subject went Off Study: ----------/----/----- (year/month/day)

INDICATE OFF STUDY REASON:

□ Study Activities Completed
□ Side effects of study intervention (complete applicable SAE form or AE Tracking Log)
□ Death
□ Subject lost to follow-up (provide comments below)
□ Subject refused follow-up (provide comments below)
□ Other (provide comments below)
□ Subject withdrew (complete Early Withdrawal section below)

EARLY WITHDRAWAL

Last Visit Completed: □ Early *Withdrawal form not completed*

Indicate the **primary** reason the subject has withdrawn from the study (select only one):
□ Subject deemed eligible but declined participation
□ Subject deemed inappropriate for study participation by the PI
□ Participant was determined to be ineligible after enrollment (provide comments below)
□ Identification of disease/condition after enrollment that warrants withdrawal
□ Unable to continue due to personal constraints
□ Side effects of study intervention
□ Other -----------------------

VISIT 4 (Week 12)

STANDARD VISIT SHEET

Visit Checklist:			
		Yes	No
1.	**Have there been any new Adverse Events?**<br>(If yes, please record in Adverse Events page)		
2.	**Have there been any changes in Concomitant Medications?**<br><br>(If yes, please record in Concomitant Medications Log)		
3.	**Laboratory data**		
4.	**Rituximab Administration**		
5.	**Is the patient experiencing a relapse?**<br><br>**(If yes record in complete physical exam form)**		
6.	**Is Adverse event check list completed?**		
7.	**Does patient withdraw or exclude from study?**		

Date (dd/mm/yy): |___|___|/|___|___|/|___|___|

VISIT 5 (Week 24)

VITAL SIGNS	
Temperature:<br><br>Respiration rate:	Blood Pressure:<br><br>Heart rate:

PHYSICAL EXAMINATION

Examine the following and place a √ in the appropriate column. If abnormal is √, then provide the condition(s) in the comments column as provided.

Body system	Normal	Abnormal	Not done	comments
Appearance				
Skin				
HEENT				
Thyroid				
Chest				
Renal system				
Cardiovascular system				
Breasts				
Heart				
Abdomen				
Musculoskeletal				
Genitalia				
Pelvic				
Rectal				
Prostate				
Vascular				
Neurological				
Lymph nodes				
specify, others				

VISIT 5 (Week 24)

CONCOMITANT MEDICATION

Has the participant used any Concomitant Medications? **No □ Yes □**, Complete below

CM No.	Medication name (Record <specify Generic or Brand> name)	Start date (year/month/day)	Stop date (year/month/day)	Or tick if ongoing at end of study?	Reason for use (Enter related AE diagnosis, or other reasons for use, e.g. Prophylaxis)	Dose (Units)	Route	Frequency
1								
2								
3								
4								
5								
6								
7								
8								
9								
10								
11								
12								
13								
14								
15								
16								

Date (dd/mm/yy): |___|___|/|___|___|/|___|___|

VISIT 5 (Week 24)

RELAPSE RECORD FORM AFTER TREATMENT

Please just mention any relapse after previous visit

Relapse	Date	Ocular Myasthenia Gravis	Generalized Myasthenia Gravis	Duration of relapse	Treatment

CORTICOSTROID`S DOSAGE CHANGE

Has administrated corticosteroid's dosage been changed?

No □ Yes □

If answer is yes, please mention the new dose----------------

PYRIDOSTIGMINE`S DOSAGE CHANGE

Has administrated pyridostigmine `s dosage been changed?

No □ Yes □

If answer is yes, please mention the new dose----------------

Date (dd/mm/yy): |___|___|/|___|___|/|___|___|

VISIT 5 (Week 24)

Evaluation of taking IVIG and Plasmapheresis:

Has patient received IVIG from the last visit?

No □ Yes □

If yes, please mention the dose and number of administrations:

Has patient undertaken plasmapheresis from the last visit?

No □ Yes □

If yes, please mention the number of this operation:

VISIT 5 (Week 24)

LABORATORY DATA

**BUN:**	**Sodium(serum):**
**Creatinine:**	**Potassium (serum):**
**AST:**	**ALP:**
**ALT:**	**Bilirubin:**
**CBC with differential:**<br>**WBC: ………….** /μL<br>**Hb: …………….** /dL<br>**HCT: ………….** %<br>**MCV: ………….** fL<br>**MCHC: ………..** g/dL<br>**PLT: ………….** /μL	

If other lab data has been taken due to patient`s condition/background diseases, please write down the corresponding lab results: ---------------

VISIT 5 (Week 24)

MONITORING PARAMETER

Subject ID:-------------

Subject number:---------------

Name of lab that test were done?----------------------

Date of test:-----------------

CD 19:-------------------------

CD 20:-------------------------

VISIT 5 (Week 24)

RITUXIMAB INFUSION FORM

Infusion date: ------------------------------ (year/ month/ day)

Place of injection: ---

Rituximab □ Induction phase □ Maintenance phase

(Please circle) week 1 2 3 4 Month ----------------

Dose of Rituximab administered on this infusion date: --------------

Was patient`s EKG taken before, during and after infusion?

□ Yes

□ No

If yes, please mention the condition of patient-----------------

If patient take antihypertensive drugs, did he/she hold the drug 12 hrs before rituximab infusion?

□ Yes

□ No

Use of premedication to prevent infusion reactions:

1) Was antihistamine drugs administered at this rituximab infusion?

No□

Yes□, please mention the name and dose: -------------------------

2) Was acetaminophen administered at this rituximab infusion?

No□

Yes□, please mention the dose: -------------------------

3) Was glucocorticoid drugs administered at this rituximab infusion?

No□

Yes□, please mention the name and dose: -------------------------

4) Was NSAIDs drugs administered at this rituximab infusion?

No□

Yes□, please mention the name and dose: -------------------------

VISIT 5 (Week 24)

Rituximab infusion speed protocol:

Total time: approximately 3 hrs

First 30 minute: 50 mg/hour (for example for 500 mg vial at 0.5 liter serum, it will be 25 ml at 30 minute that it equals 20 drops per min)

Then increased by 50 mg/hour every 30 minutes to a maximum of 400 mg/hour

Was the rituximab infusion administered completely?

□Yes □No

If No, please indicate:

Nurse name and signature:

VISIT 5 (Week 24)

RITUXIMAB INFUSION REACTION

Infusion Reaction:

No □ Yes □

If answer is yes, please complete details:

Time of reaction start:

□ Within 30 min

□ Within 30min-2hrs

□ After 2 hrs

□ After 24 hrs

Signs and symptoms of infusion reactions:

□ Fever and/or shaking chills
□ Flushing and/or itching
□ Alterations in heart rate and blood pressure
□ Dyspnea or chest discomfort
□ Back or abdominal pain
□ Nausea, vomiting, and/or diarrhea
□ Various types of skin rashes
□ others:--------

Classification of infusion reactions:

□ **Grade 1.** Mild transient reaction; infusion interruption not indicated; intervention not indicated

□ **Grade 2.** Therapy or infusion interruption indicated but responds promptly to symptomatic treatment (eg, antihistamines, NSAIDs, narcotics, intravenous fluids); prophylactic medication indicated for less than or equal to 24 hours

□ **Grade 3.** Prolonged (ie, not rapidly responsive to symptomatic medication and/or brief interruption of infusion); recurrence of symptoms following initial improvement; hospitalization indicated for other clinical sequelae

□ **Grade 4.** Life-threatening consequences; urgent intervention indicated

□ **Grade 5.** Death

Is anaphylactic reaction happened?

Yes □ No □

Repeated premedication after or during infusion reaction:

Yes □ No □

Treatment and dosage:

Does infusion temporarily interrupted and then continued?-----------------

Does infusion rate reduced and completed (mention the speed)?----------------

Does infusion rate reduced and not completed?---------------

VISIT 5 (Week 24)

Visit:-------

Current date:--------

Subject ID:-------------, Subject number:-------------

Enter score here:----------------------

پرسشنامه کیفیت زندگی میاستنی گراویس(MG-QOL15):

لطفا مشخص کنید هر جمله تا چه حد درست است (در چند هفته قبل).

	هرگز	کمی	تا حدودی	زیاد	بسیار زیاد
	۰	۱	۲	۳	۴
۱. من از بیماری میاستنی ام خسته شدم.					
۲. من در استفاده از چشمانم مشکل دارم.					
۳. من به علت بیماری میاستنی در غذا خوردن مشکل دارم.					
۴. من به علت بیماری میاستنی ام، فعالیتهای اجتماعی ام را محدود کردم.					
۵. بیماری میاستنی توانایی من را برای لذت بردن از سرگرمی ها محدود می کند.					
۶. من به علت بیماری میاستنی ام در برآورده کردن نیازهای خانواده ام مشکل دارم.					
۷. من باید برای بیماری میاستنی خود چاره ای بیاندیشم.					
۸. بیماری میاستنی بر روی مهارتهای کاری و موقعیت شغلی من تاثیر منفی داشته است.					
۹. من به علت بیماری میاستنی ام در صحبت کردن مشکل دارم.					

					۱۰. من به علت بیماری میاستنی ام در رانندگی مشکل دارم.
					۱۱. من از بابت بیماری میاستنی ام افسرده هستم.
					۱۲. من به علت بیماری میاستنی ام در راه رفتن مشکل دارم.
					۱۳. من به علت بیماری میاستنی ام برای حضور در اماکن عمومی مشکل دارم.
					۱۴. بیماری میاستنی زندگی من را تحت الشعاع قرار داده است.
					۱۵. من در انجام نظافت شخصی ام مشکل دارم.

	**نمره کل**

VISIT 5 (Week 24)

Visit:-------

Current date:--------

Subject ID:-------------,	Subject number:--------------

Enter score here:-----------------------

MG Activities of Daily Living Scale: (MG-ADL)

Grade	0	1	2	3	Score (0, 1, 2, 3)
**Talking**	Normal	Intermittent slurring of nasal speech	Constant slurring or nasal, but can be understood	Difficult to understand speech	
**Chewing**	Normal	Fatigue with solid food	Fatigue with soft food	Gastric tube	
**Swallowing**	Normal	Rare episode of choking	Frequent choking necessitating	Gastric tube	
**Breathing**	Normal	Shortness of breath with exertion	Shortness of breath at rest	Ventilator dependence	
**Impairment of ability to brush teeth or comb hair**	none	Extra effort, but no rest periods needed	Rest periods needed	Cannot do one of these functions	
**Impairment of ability to arise from a chair**	none	Mild, sometimes uses arms	Moderate, always uses arms	Severe, requires assistance	
**Double vision**	none	Occurs, but not daily	Occurs, but not daily	Constant	
**Eyelid droop**	None	Occurs, but not daily	Daily, but not constant	Constant	

Total score	

VISIT 5 (Week 24)

Visit:-------

Current date:-------- Subject ID:------------,

Subject number:-------------

Enter score here:----------------------

**MGFA Clinical Classification**	
**Class I**	Any ocular muscle weakness<br>May have weakness of eye closure<br>Strength of all the other muscles is normal
**Class II**	Mild weakness affecting muscles other than ocular muscles<br>May also have ocular muscle weakness of any severity
**IIa**	Predominantly affecting limb, axial muscles, or both.<br>May also have lesser involvement of oropharyngeal muscles
**IIb**	Predominantly affecting oropharyngeal, respiratory muscles, or both<br>May also have lesser or equal involvement of limb, axial muscles, or both
**Class III**	Moderate weakness affecting muscles other than ocular muscles<br>May also have ocular muscle weakness of any severity
**IIIa**	Predominantly affecting limb, axial muscles, or both<br>May also have lesser involvement of oropharyngeal muscles
**IIIb**	Predominantly affecting oropharyngeal, respiratory muscles, or both<br>May also have lesser or equal involvement of limb, axial muscles, or both
**Class IV**	Severe weakness affecting muscles other than ocular muscles<br>May also have ocular muscle weakness of any severity
**IVa**	Predominantly affecting limb, axial muscles, or both<br>May also have lesser involvement of oropharyngeal muscles
**IVb**	Predominantly affecting oropharyngeal, respiratory muscles, or both<br>May also have lesser or equal involvement of limb, axial muscles, or both
**Class V**	Defined as intubation, with or without mechanical ventilation, except when employed during routine postoperative management. The use of a feeding tube without intubation places the patient in class IVb.

VISIT 5 (Week 24)

Visit:-------

Current date:--------

Subject ID:-------------, Subject number:--------------

The Myasthenia gravis Composite scale (MG Composite)

**Ptosis, upward (physician examination)**	$> 45\ seconds = 0$	11-45 seconds = 1	1-10 seconds = 2	Immediate = 3
**Double vision on lateral gaze, left or right (physician examination)**	$> 45\ seconds = 0$	11-45 seconds = 1	1-10 seconds = 3	Immediate = 4
**Eye closure (physician examination)**	Normal = 0	Mild weakness (can be forced open with effort) = 0	Moderate weakness (can be forced open easily) = 1	Severe weakness (unable to keep eye closed) = 2
**Talking (patient history)**	Normal = 0	Intermittent slurring or nasal speech = 2	Constant slurring or nasal but can be understood = 4	Difficult to understand speech = 6
**Chewing (patient history)**	Normal = 0	Fatigue with solid food = 2	Fatigue with solid food = 4	Gastric tube = 6
**Swallowing (patient history)**	Normal = 0	Rare episode of choking or trouble = 2	Frequent trouble swallowing e.g. necessitating changes in diet = 5	Gastric tube = 6
**Breathing (thought to be caused by MG)**	Normal = 0	Shortness of breath with exertion = 2	Shortness of breath at rest = 4	Ventilator dependence = 9
**Neck flexion or extension (weakest) (physician examination)**	Normal = 0	Mild weakness = 1	Moderate weakness (i.e. $\sim 50\% weak \pm 15\%) = 3^a$	Severe weakness = 4
**Shoulder abduction (physician examination)**	Normal = 0	Mild weakness = 2	Moderate weakness (i.e. $\sim 50\% weak \pm 15\%) = 4^a$	Severe weakness = 5
**Hip flexion (physician examination)**	Normal = 0	Mild weakness = 2	Moderate weakness (i.e. $\sim 50\% weak \pm 15\%) = 4^a$	Severe weakness = 5

[a] Moderate weakness for neck and limb items should be construed as weakness that equals roughly 50% _15% of expected normal strength. Any weakness milder than that would be mild and any weakness more severe than that would be classified as severe.

Total score	

<u>VISIT 5 (Week 24)</u>

Please specify the MGFA Post intervention Status:

Complete Stable Remission (CSR): The patient has had no symptoms or signs of MG for at least 1 year and has received no therapy for MG during that time. There is no weakness of any muscle on careful examination by someone skilled in the evaluation of neuromuscular disease. Isolated weakness of eyelid closure is accepted.

Pharmacologic Remission (PR): The same criteria as for **CSR** except that the patient continues to take some form of therapy for MG. Patients taking cholinesterase inhibitors are excluded from this category because their use suggests the presence of weakness.

Minimal Manifestations (MM): The patient has no symptoms of functional limitations from MG but has some weakness on examination of some muscles. This class recognizes that some patients who otherwise meet the definition of **CSR** or **PR** do have weakness that is only detectable by careful examination.

MM-0: The patient has received no MG treatment for at least 1 year.

MM-1: The patient continues to receive some form of immunosuppression but no cholinesterase inhibitors or other symptomatic therapy.

MM-2: The patient has received only low-dose cholinesterase inhibitors (<120 mg pyridostigmine/day) for at least 1 year.

MM-3: The patient has received cholinesterase inhibitors or other symptomatic therapy and some form of immunosuppression during the past year.

Change in Status

Improved (I): A substantial decrease in pretreatment clinical manifestations or a sustained substantial reduction in MG medications as defined in the protocol. In prospective studies, this should be defined as a specific decrease in QMG score.

Unchanged (U): No substantial change in pretreatment clinical manifestations or reduction in MG medications as defined in the protocol. In prospective studies, this should be defined in terms of maximum change in QMG score.

Worse (W): A substantial increase in pretreatment clinical manifestations or substantial increase in MG medications as defined in the protocol. In prospective studies, this should be defined as a specific increase in QMG score.

Exacerbation (E): Patients who have fulfilled criteria of **CSR**, **PR** or **MM** but subsequently developed clinical findings greater than permitted by these criteria.

Died of MG (D of MG): Patients who died of MG, of complications of MG therapy or within 30 days after thymectomy.

VISIT 5 (Week 24)

SERIOUS ADVERSE EVENTS Page 1 of 2

Event Start Date: --------/----/---- (year/month/day), Event end Date: --------/----/---- (year/month/day)

Date Reported: --------/----/---- (year/month/day), Reported to staff by:--------------------------------

Death Date (If applicable): --------/----/---- (year/month/day)

Death Occurred (check one):

□ Within 24 hours of investigational therapy □ Within 30 days of investigational therapy

□ Within 7 days of investigational therapy □ After 30 days of investigational therapy

Did the SAE occur at your site or at a site for which the PI is responsible? □ Yes □No

SAE Description/Narrative:

Treating Physician Comments:

PI Comments:

Outcome: (check one)

□ Fatal/Died
□ Intervention for AE Continuing
□ Not Recovered/Not Resolved
□ Recovered/Resolved with Sequelae
□ Recovered/Resolved without Sequelae
□ Recovering/Resolving

Consent Form Change Required? □ Yes □ No

SAE Classification: (check all that apply)

□ Fatal (resulted in death)
□ A life-threatening occurrence
□ Requires inpatient hospitalization or prolongation of existing hospitalization
□ Results in persistent or significant disability/incapacity
□ Results in congenital anomaly/birth defect
□ A significant medical incident that, based upon appropriate medical judgment, may jeopardize the subject and require medical or surgical intervention to prevent one of the outcomes listed above.
□ Loss of confidentiality that results in criminal or civil liability for participation or damage to financial standing, employability, insurability or reputation of the participant

VISIT 5 (Week 24)

SERIOUS ADVERSE EVENTS Page 2 of 2

SAE Reported Symptom:

Category: [refer to the Safety Profiler website to search the Category and Toxicity of the SAE symptom reported: http://safetyprofiler-ctep.nci.nih.gov/CTC/CTC.aspx]

Toxicity:

Grade/Severity (check one):

□ 1 - Mild
□ 2 - Moderate
□ 3 - Severe
□ 4 - Life Threatening
□ 5 – Death (Fatal)

Unexpected? □ Yes □No

Dose Limiting Toxicity (DLT)? □ Yes □ No □ Not Applicable

Action taken:

□ Dose reduced

□ Dose interrupted, then reduced

□ None

□ Regimen interrupted

□ Therapy discontinued

Primary attribution: (check one)

□ Definite

□ Probable

□ Possible

□ Unlikely

□ Unrelated

Detailed Attribution: (check one)

□ **Disease/Condition** Specify:

□ **Investigational Treatment** Specify:

□ **Non-investigational Treatment** Specify:

□ **Other** Specify:

VISIT 5 (Week 24)

ADVERSE EVENT CHECK LIST

Head and Lymphatic System	Yes	No
Lymphopenia		
Leukopenia		
Neutropenia		
Thrombocytopenia		
Anemia		

Musculoskeletal	Yes	No
Myalgia		
Arthralgia		

	Yes	No
Infusion reaction		

Nervous System	Yes	No
Dizziness		
Anxiety		
Headache		
Progressive multifocal leukoencephalopathy		

Digestive System	Yes	No
Nausea		
Diarrhea		
Vomiting		

Cardiovascular System	Yes	No
Hypotension		
Hypertension		

Skin and Appendages	Yes	No
Night sweats		
Rash		
Pruritus		
Urticaria		

Body as a Whole	Yes	No
Fever		
Chills		
Infection		
Asthenia		
Abdominal Pain		
Pain		
Back Pain		
Throat Irritation		

Metabolic and Nutritional Disorder	Yes	No
Angioedema		
Hyperglycemia		
Peripheral Edema		

VISIT 5 (Week 24)

ADVERSE EVENTS

	**Event Name** (Please give Diagnosis if known)	**Start date** (year/month/day)	**Stop date** (year/month/day)	**Serious?** If serious, please complete a JRO SAE form	**Con-comitant Medication given**	**Severity** 0 - Mild 1- Mode-rate 2 - Severe	**Study Drug Action** 0 - None 1 - Temporarily Interrupted 2 - permanently withdrawn	**Outcome** 0 - Resolved 1- Resolved with sequelea 2 - Not resolved	**Relationship to Study Drug** 0 - Definitely 1 - Probably 2 - Possibly 3 - Unlikely 4 - Not related 5 - Not assessable
1.				□ No □ Yes	□ No □ Yes				
2.				□ No □ Yes	□ No □ Yes				
3.				□ No □ Yes	□ No □ Yes				
4.				□ No □ Yes	□ No □ Yes				
5.				□ No □ Yes	□ No □ Yes				
6.				□ No □ Yes	□ No □ Yes				
7.				□ No □ Yes	□ No □ Yes				
8.				□ No □ Yes	□ No □ Yes				
9.				□ No □ Yes	□ No □ Yes				

Date (dd/mm/yy): |___|___|/|___|___|/|___|___|

10.				□ No<br>□ Yes	□ No<br>□ Yes				
11.				□ No<br>□ Yes	□ No<br>□ Yes				
12.				□ No<br>□ Yes	□ No<br>□ Yes				
13.				□ No<br>□ Yes	□ No<br>□ Yes				
14.				□ No<br>□ Yes	□ No<br>□ Yes				
15.				□ No<br>□ Yes	□ No<br>□ Yes				
16.				□ No<br>□ Yes	□ No<br>□ Yes				
17.				□ No<br>□ Yes	□ No<br>□ Yes				
18.				□ No<br>□ Yes	□ No<br>□ Yes				

I have reviewed the AEs on this page and have assessed them for seriousness, causality, severity and outcome and confirm that, to the best of my knowledge, it accurately reflects the study information obtained for this participant
PI signature ________________________________ _________________ Date:_________________________ Please check box if this is the last page used

VISIT 5 (Week 24)

SUBJECT OFF STUDY

Date subject went Off Study: ----------/----/----- (year/month/day)

INDICATE OFF STUDY REASON:

□ Study Activities Completed
□ Side effects of study intervention (complete applicable SAE form or AE Tracking Log)
□ Death
□ Subject lost to follow-up (provide comments below)
□ Subject refused follow-up (provide comments below)
□ Other (provide comments below)
□ Subject withdrew (complete Early Withdrawal section below)

EARLY WITHDRAWAL

Last Visit Completed: □ Early *Withdrawal form not completed*

Indicate the **primary** reason the subject has withdrawn from the study (select only one):
□ Subject deemed eligible but declined participation
□ Subject deemed inappropriate for study participation by the PI
□ Participant was determined to be ineligible after enrollment (provide comments below)
□ Identification of disease/condition after enrollment that warrants withdrawal
□ Unable to continue due to personal constraints
□ Side effects of study intervention
□ Other -----------------------

VISIT 5 (Week 24)

STANDARD VISIT SHEET

Visit Checklist:			
		Yes	No
1.	**Have there been any new Adverse Events?**<br>(If yes, please record in Adverse Events page)		
2.	**Have there been any changes in Concomitant Medications?**<br><br>(If yes, please record in Concomitant Medications Log)		
3.	**Laboratory data**		
4.	**Rituximab Administration**		
5.	**Is the patient experiencing a relapse?**<br><br>**(If yes record in complete physical exam form)**		
6.	**Is Adverse event check list completed?**		
7.	**Does patient withdraw or exclude from study?**		

VISIT 6 (Week 36)

VITAL SIGNS	
Temperature:<br><br>Respiration rate:	Blood Pressure:<br><br>Heart rate:

PHYSICAL EXAMINATION

Examine the following and place a √ in the appropriate column. If abnormal is √, then provide the condition(s) in the comments column as provided.

Body system	Normal	Abnormal	Not done	comments
Appearance				
Skin				
HEENT				
Thyroid				
Chest				
Renal system				
Cardiovascular system				
Breasts				
Heart				
Abdomen				
Musculoskeletal				
Genitalia				
Pelvic				
Rectal				
Prostate				
Vascular				
Neurological				
Lymph nodes				
specify, others				

VISIT 6 (Week 36)

CONCOMITANT MEDICATION

Has the participant used any Concomitant Medications? **No □ Yes □,** Complete below

CM No.	Medication name (Record <specify Generic or Brand> name)	Start date (year/month/day)	Stop date (year/month/day)	Or tick if ongoing at end of study?	Reason for use (Enter related AE diagnosis, or other reasons for use, e.g. Prophylaxis)	Dose (Units)	Route	Frequency
1								
2								
3								
4								
5								
6								
7								
8								
9								
10								
11								
12								
13								
14								
15								
16								

Date (dd/mm/yy): |___|___|/|___|___|/|___|___|

VISIT 6 (Week 36)

RELAPSE RECORD FORM AFTER TREATMENT

Please just mention any relapse after previous visit

Relapse	Date	Ocular Myasthenia Gravis	Generalized Myasthenia Gravis	Duration of relapse	Treatment

CORTICOSTROID`S DOSAGE CHANGE

Has administrated corticosteroid's dosage been changed?

No □ Yes □

If answer is yes, please mention the new dose----------------

PYRIDOSTIGMINE`S DOSAGE CHANGE

Has administrated pyridostigmine `s dosage been changed?

No □ Yes □

If answer is yes, please mention the new dose----------------

VISIT 6 (Week 36)

Evaluation of taking IVIG and Plasmapheresis:

Has patient received IVIG from the last visit?

No □ Yes □

If yes, please mention the dose and number of administrations:

Has patient undertaken plasmapheresis from the last visit?

No □ Yes □

If yes, please mention the number of this operation:

VISIT 6 (Week 36)

LABORATORY DATA

**BUN:**	**Sodium(serum):**
**Creatinine:**	**Potassium (serum):**
**AST:**	**ALP:**
**ALT:**	**Bilirubin:**
**CBC with differential:**<br>**WBC: ………….** /μL<br>**Hb: …………….** /dL<br>**HCT: ………….** %<br>**MCV: ………….** fL<br>**MCHC: ………..** g/dL<br>**PLT: ………….** /μL	

If other lab data has been taken due to patient`s condition/background diseases, please write down the corresponding lab results: ---------------

Date (dd/mm/yy): |___|___|/|___|___|/|___|___|

VISIT 6 (Week 36)

Visit:-------

Current date:--------

Subject ID:------------, Subject number:-------------

Enter score here:----------------------

پرسشنامه کیفیت زندگی میاستنی گراویس(MG-QOL15):

لطفا مشخص کنید هر جمله تا چه حد درست است (در چند هفته قبل).

	هرگز	کمی	تا حدودی	زیاد	بسیار زیاد
	۰	۱	۲	۳	۴
۱. من از بیماری میاستنی ام خسته شدم.					
۲. من در استفاده از چشمانم مشکل دارم.					
۳. من به علت بیماری میاستنی در غذا خوردن مشکل دارم.					
۴. من به علت بیماری میاستنی ام، فعالیتهای اجتماعی ام را محدود کردم.					
۵. بیماری میاستنی توانایی من را برای لذت بردن از سرگرمی ها محدود می کند.					
۶. من به علت بیماری میاستنی ام در برآورده کردن نیازهای خانواده ام مشکل دارم.					
۷. من باید برای بیماری میاستنی خود چاره ای بیاندیشم.					
۸. بیماری میاستنی بر روی مهارتهای کاری و موقعیت شغلی من تاثیر منفی داشته است.					
۹. من به علت بیماری میاستنی ام در صحبت کردن مشکل دارم.					

					۱۰. من به علت بیماری میاستنی ام در رانندگی مشکل دارم.
					۱۱. من از بابت بیماری میاستنی ام افسرده هستم.
					۱۲. من به علت بیماری میاستنی ام در راه رفتن مشکل دارم.
					۱۳. من به علت بیماری میاستنی ام برای حضور در اماکن عمومی مشکل دارم.
					۱۴. بیماری میاستنی زندگی من را تحت الشعاع قرار داده است.
					۱۵. من در انجام نظافت شخصی ام مشکل دارم.

	**نمره کل**

VISIT 6 (Week 36)

Visit:-------

Current date:--------

Subject ID:-------------, Subject number:--------------

Enter score here:-----------------------

MG Activities of Daily Living Scale: (MG-ADL)

Grade	0	1	2	3	Score (0, 1, 2, 3)
**Talking**	Normal	Intermittent slurring of nasal speech	Constant slurring or nasal, but can be understood	Difficult to understand speech	
**Chewing**	Normal	Fatigue with solid food	Fatigue with soft food	Gastric tube	
**Swallowing**	Normal	Rare episode of choking	Frequent choking necessitating	Gastric tube	
**Breathing**	Normal	Shortness of breath with exertion	Shortness of breath at rest	Ventilator dependence	
**Impairment of ability to brush teeth or comb hair**	none	Extra effort, but no rest periods needed	Rest periods needed	Cannot do one of these functions	
**Impairment of ability to arise from a chair**	none	Mild, sometimes uses arms	Moderate, always uses arms	Severe, requires assistance	
**Double vision**	none	Occurs, but not daily	Occurs, but not daily	Constant	
**Eyelid droop**	None	Occurs, but not daily	Daily, but not constant	Constant	

Total score	

VISIT 6 (Week 36)

Visit:-------

Current date:-------- Subject ID:------------,

Subject number:-------------

Enter score here:----------------------

MGFA Clinical Classification	
**Class I**	Any ocular muscle weakness<br>May have weakness of eye closure<br>Strength of all the other muscles is normal
**Class II**	Mild weakness affecting muscles other than ocular muscles<br>May also have ocular muscle weakness of any severity
**IIa**	Predominantly affecting limb, axial muscles, or both.<br>May also have lesser involvement of oropharyngeal muscles
**IIb**	Predominantly affecting oropharyngeal, respiratory muscles, or both<br>May also have lesser or equal involvement of limb, axial muscles, or both
**Class III**	Moderate weakness affecting muscles other than ocular muscles<br>May also have ocular muscle weakness of any severity
**IIIa**	Predominantly affecting limb, axial muscles, or both<br>May also have lesser involvement of oropharyngeal muscles
**IIIb**	Predominantly affecting oropharyngeal, respiratory muscles, or both<br>May also have lesser or equal involvement of limb, axial muscles, or both
**Class IV**	Severe weakness affecting muscles other than ocular muscles<br>May also have ocular muscle weakness of any severity
**IVa**	Predominantly affecting limb, axial muscles, or both<br>May also have lesser involvement of oropharyngeal muscles
**IVb**	Predominantly affecting oropharyngeal, respiratory muscles, or both<br>May also have lesser or equal involvement of limb, axial muscles, or both
**Class V**	Defined as intubation, with or without mechanical ventilation, except when employed during routine postoperative management. The use of a feeding tube without intubation places the patient in class IVb.

<u>VISIT 6 (Week 36)</u>

Visit:-------

Current date:--------

Subject ID:------------, Subject number:-------------

The Myasthenia gravis Composite scale (MG Composite)

**Ptosis, upward (physician examination)**	$> 45\ seconds$ = 0	11-45 seconds = 1	1-10 seconds = 2	Immediate = 3
**Double vision on lateral gaze, left or right (physician examination)**	$> 45\ seconds$ = 0	11-45 seconds = 1	1-10 seconds = 3	Immediate = 4
**Eye closure (physician examination)**	Normal = 0	Mild weakness (can be forced open with effort) = 0	Moderate weakness (can be forced open easily) = 1	Severe weakness (unable to keep eye closed) = 2
**Talking (patient history)**	Normal = 0	Intermittent slurring or nasal speech = 2	Constant slurring or nasal but can be understood = 4	Difficult to understand speech = 6
**Chewing (patient history)**	Normal = 0	Fatigue with solid food = 2	Fatigue with solid food = 4	Gastric tube = 6
**Swallowing (patient history)**	Normal = 0	Rare episode of choking or trouble = 2	Frequent trouble swallowing e.g. necessitating changes in diet = 5	Gastric tube = 6
**Breathing (thought to be caused by MG)**	Normal = 0	Shortness of breath with exertion = 2	Shortness of breath at rest = 4	Ventilator dependence = 9
**Neck flexion or extension (weakest) (physician examination)**	Normal = 0	Mild weakness = 1	Moderate weakness (i.e. $\sim 50\% weak \pm 15\%$) = 3[a]	Severe weakness = 4
**Shoulder abduction (physician examination)**	Normal = 0	Mild weakness = 2	Moderate weakness (i.e. $\sim 50\% weak \pm 15\%$) = 4[a]	Severe weakness = 5
**Hip flexion (physician examination)**	Normal = 0	Mild weakness = 2	Moderate weakness (i.e. $\sim 50\% weak \pm 15\%$) = 4[a]	Severe weakness = 5

[a] Moderate weakness for neck and limb items should be construed as weakness that equals roughly 50% _15% of expected normal strength. Any weakness milder than that would be mild and any weakness more severe than that would be classified as severe.

Total score	

VISIT 6 (Week 36)

SERIOUS ADVERSE EVENTS Page 1 of 2

Event Start Date: --------/----/---- (year/month/day), Event end Date: --------/----/---- (year/month/day)

Date Reported: --------/----/---- (year/month/day), Reported to staff by:---------------------------------

Death Date (If applicable): --------/----/---- (year/month/day)

Death Occurred (check one):

□ Within 24 hours of investigational therapy

□ Within 7 days of investigational therapy

□ Within 30 days of investigational therapy

□ After 30 days of investigational therapy

Did the SAE occur at your site or at a site for which the PI is responsible? □ Yes □No

SAE Description/Narrative:

Treating Physician Comments:

PI Comments:

Outcome: (check one)

□ Fatal/Died
□ Intervention for AE Continuing
□ Not Recovered/Not Resolved
□ Recovered/Resolved with Sequelae
□ Recovered/Resolved without Sequelae
□ Recovering/Resolving

Consent Form Change Required? □ Yes □ No

SAE Classification: (check all that apply)

□ Fatal (resulted in death)
□ A life-threatening occurrence
□ Requires inpatient hospitalization or prolongation of existing hospitalization
□ Results in persistent or significant disability/incapacity
□ Results in congenital anomaly/birth defect
□ A significant medical incident that, based upon appropriate medical judgment, may jeopardize the subject and require medical or surgical intervention to prevent one of the outcomes listed above.
□ Loss of confidentiality that results in criminal or civil liability for participation or damage to financial standing, employability, insurability or reputation of the participant

SERIOUS ADVERSE EVENTS Page 2 of 2

SAE Reported Symptom:

Category: [refer to the Safety Profiler website to search the Category and Toxicity of the SAE symptom reported: http://safetyprofiler-ctep.nci.nih.gov/CTC/CTC.aspx]

Toxicity:

Grade/Severity (check one):
□ 1 - Mild
□ 2 - Moderate
□ 3 - Severe
□ 4 - Life Threatening
□ 5 – Death (Fatal)

Unexpected? □ Yes □No

Dose Limiting Toxicity (DLT)? □ Yes □ No □ Not Applicable

Action taken:

□ Dose reduced

□ Dose interrupted, then reduced

□ None

□ Regimen interrupted

□ Therapy discontinued

Primary attribution: (check one)

□ Definite

□ Probable

□ Possible

□ Unlikely

□ Unrelated

Detailed Attribution: (check one)

□ **Disease/Condition** Specify:

□ **Investigational Treatment** Specify:

□ **Non-investigational Treatment** Specify:

□ **Other** Specify:

Date (dd/mm/yy): |___|___|/|___|___|/|___|___|

VISIT 6 (Week 36)

ADVERSE EVENT CHECK LIST

Head and Lymphatic System	Yes	No
Lymphopenia		
Leukopenia		
Neutropenia		
Thrombocytopenia		
Anemia		

Digestive System	Yes	No
Nausea		
Diarrhea		
Vomiting		

Musculoskeletal	Yes	No
Myalgia		
Arthralgia		

Nervous System	Yes	No
Dizziness		
Anxiety		
Headache		
Progressive multifocal leukoencephalopathy		

Skin and Appendages	Yes	No
Night sweats		
Rash		
Pruritus		
Urticaria		

Metabolic and Nutritional Disorder	Yes	No
Angioedema		
Hyperglycemia		
Peripheral Edema		

Cardiovascular System	Yes	No
Hypotension		
Hypertension		

Body as a Whole	Yes	No
Fever		
Chills		
Infection		
Asthenia		
Abdominal Pain		
Pain		
Back Pain		
Throat Irritation		

	Yes	No
Infusion reaction		

Date (dd/mm/yy): |___|___|/|___|___|/|___|___|

VISIT 6 (Week 36)

ADVERSE EVENTS

	**Event Name** (Please give Diagnosis if known)	**Start date** (year/month/day)	**Stop date** (year/month/day)	**Serious?** If serious, please complete a JRO SAE form	**Con-comitant Medication given**	**Severity** 0 - Mild 1- Mode-rate 2 - Severe	**Study Drug Action** 0 - None 1 - Temporarily Interrupted 2 - permanently withdrawn	**Outcome** 0 - Resolved 1- Resolved with sequelea 2 - Not resolved	**Relationship to Study Drug** 0 - Definitely 1 - Probably 2 - Possibly 3 - Unlikely 4 - Not related 5 - Not assessable
1.				□ No □ Yes	□ No □ Yes				
2.				□ No □ Yes	□ No □ Yes				
3.				□ No □ Yes	□ No □ Yes				
4.				□ No □ Yes	□ No □ Yes				
5.				□ No □ Yes	□ No □ Yes				
6.				□ No □ Yes	□ No □ Yes				
7.				□ No □ Yes	□ No □ Yes				
8.				□ No □ Yes	□ No □ Yes				
9.				□ No □ Yes	□ No □ Yes				

Date (dd/mm/yy): |___|___|/|___|___|/|___|___|

10.				□ No<br>□ Yes	□ No<br>□ Yes				
11.				□ No<br>□ Yes	□ No<br>□ Yes				
12.				□ No<br>□ Yes	□ No<br>□ Yes				
13.				□ No<br>□ Yes	□ No<br>□ Yes				
14.				□ No<br>□ Yes	□ No<br>□ Yes				
15.				□ No<br>□ Yes	□ No<br>□ Yes				
16.				□ No<br>□ Yes	□ No<br>□ Yes				
17.				□ No<br>□ Yes	□ No<br>□ Yes				
18.				□ No<br>□ Yes	□ No<br>□ Yes				

I have reviewed the AEs on this page and have assessed them for seriousness, causality, severity and outcome and confirm that, to the best of my knowledge, it accurately reflects the study information obtained for this participant

PI signature ________________________________ __________________ Date:__________________________ Please check box if this is the last page used

VISIT 6 (Week 36)

SUBJECT OFF STUDY

Date subject went Off Study: ----------/----/----- (year/month/day)

INDICATE OFF STUDY REASON:

□ Study Activities Completed
□ Side effects of study intervention (complete applicable SAE form or AE Tracking Log)
□ Death
□ Subject lost to follow-up (provide comments below)
□ Subject refused follow-up (provide comments below)
□ Other (provide comments below)
□ Subject withdrew (complete Early Withdrawal section below)

EARLY WITHDRAWAL

Last Visit Completed: □ Early *Withdrawal form not completed*

Indicate the **primary** reason the subject has withdrawn from the study (select only one):
□ Subject deemed eligible but declined participation
□ Subject deemed inappropriate for study participation by the PI
□ Participant was determined to be ineligible after enrollment (provide comments below)
□ Identification of disease/condition after enrollment that warrants withdrawal
□ Unable to continue due to personal constraints
□ Side effects of study intervention
□ Other -----------------------

VISIT 6 (Week 36)

STANDARD VISIT SHEET

Visit Checklist:			
		Yes	No
1.	**Have there been any new Adverse Events?**<br>(If yes, please record in Adverse Events page)		
2.	**Have there been any changes in Concomitant Medications?**<br>(If yes, please record in Concomitant Medications Log)		
3.	**Laboratory data**		
4.	**Rituximab Administration**		
5.	**Is the patient experiencing a relapse?**<br>**(If yes record in complete physical exam form)**		
6.	**Is Adverse event check list completed?**		
7.	**Does patient withdraw or exclude from study?**		

VISIT 7 (Week 48)

VITAL SIGNS	
Temperature:<br><br>Respiration rate:	Blood Pressure:<br><br>Heart rate:

PHYSICAL EXAMINATION

Examine the following and place a √ in the appropriate column. If abnormal is √, then provide the condition(s) in the comments column as provided.

Body system	Normal	Abnormal	Not done	comments
Appearance				
Skin				
HEENT				
Thyroid				
Chest				
Renal system				
Cardiovascular system				
Breasts				
Heart				
Abdomen				
Musculoskeletal				
Genitalia				
Pelvic				
Rectal				
Prostate				
Vascular				
Neurological				
Lymph nodes				
specify, others				

VISIT 7 (Week 48)

CONCOMITANT MEDICATION

Has the participant used any Concomitant Medications? **No □ Yes □**, Complete below

CM No.	Medication name (Record <specify Generic or Brand> name)	Start date (year/month/day)	Stop date (year/month/day)	Or tick if ongoing at end of study?	Reason for use (Enter related AE diagnosis, or other reasons for use, e.g. Prophylaxis)	Dose (Units)	Route	Frequency
1								
2								
3								
4								
5								
6								
7								
8								
9								
10								
11								
12								
13								
14								
15								
16								

Date (dd/mm/yy): |___|___|/|___|___|/|___|___|

<u>VISIT 7 (Week 48)</u>

RELAPSE RECORD FORM AFTER TREATMENT

Please <u>just</u> mention any relapse after previous visit

Relapse	Date	Ocular Myasthenia Gravis	Generalized Myasthenia Gravis	Duration of relapse	Treatment

CORTICOSTROID`S DOSAGE CHANGE

Has administrated corticosteroid's dosage been changed?

No □ Yes □

If answer is yes, please mention the new dose----------------

PYRIDOSTIGMINE`S DOSAGE CHANGE

Has administrated pyridostigmine `s dosage been changed?

No □ Yes □

If answer is yes, please mention the new dose----------------

Date (dd/mm/yy): |___|___|/|___|___|/|___|___|

VISIT 7 (Week 48)

Evaluation of taking IVIG and Plasmapheresis:

Has patient received IVIG from the last visit?

No □ Yes □

If yes, please mention the dose and number of administrations:

Has patient undertaken plasmapheresis from the last visit?

No □ Yes □

If yes, please mention the number of this operation:

MONITORING PARAMETER

Subject ID:-------------

Subject number:---------------

Name of lab that test were done?----------------------

Date of test:-----------------

Anti-MuSK antibody:----------------------

Anti-AChR antibody:----------------------

VISIT 7 (Week 48)

LABORATORY DATA

**BUN:**	**Sodium(serum):**
**Creatinine:**	**Potassium (serum):**
**AST:**	**ALP:**
**ALT:**	**Bilirubin:**
**CBC with differential:**<br>**WBC: ………….** /µL<br>**Hb: …………….** /dL<br>**HCT: ………….** %<br>**MCV: ………….** fL<br>**MCHC: ………..** g/dL<br>**PLT: ………….** /µL	

If other lab data has been taken due to patient`s condition/background diseases please write down the corresponding lab results: --------------

Date (dd/mm/yy): |___|___|/|___|___|/|___|___|

VISIT 7 (Week 48)

Visit:-------

Current date:--------

Subject ID:-------------,	Subject number:-------------

Enter score here:-----------------------

پرسشنامه کیفیت زندگی میاستنی گراویس(MG-QOL15):

لطفا مشخص کنید هر جمله تا چه حد درست است (در چند هفته قبل).

	هرگز	کمی	تا حدودی	زیاد	بسیار زیاد
	۰	۱	۲	۳	۴
۱. من از بیماری میاستنی ام خسته شدم.					
۲. من در استفاده از چشمانم مشکل دارم.					
۳. من به علت بیماری میاستنی در غذا خوردن مشکل دارم.					
۴. من به علت بیماری میاستنی ام، فعالیتهای اجتماعی ام را محدود کردم.					
۵. بیماری میاستنی توانایی من را برای لذت بردن از سرگرمی ها محدود می کند.					
۶. من به علت بیماری میاستنی ام در برآورده کردن نیازهای خانواده ام مشکل دارم.					
۷. من باید برای بیماری میاستنی خود چاره ای بیاندیشم.					
۸. بیماری میاستنی بر روی مهارتهای کاری و موقعیت شغلی من تاثیر منفی داشته است.					
۹. من به علت بیماری میاستنی ام در صحبت کردن مشکل دارم.					

۱۰. من به علت بیماری میاستنی ام در رانندگی مشکل دارم.					
۱۱. من از بابت بیماری میاستنی ام افسرده هستم.					
۱۲. من به علت بیماری میاستنی ام در راه رفتن مشکل دارم.					
۱۳. من به علت بیماری میاستنی ام برای حضور در اماکن عمومی مشکل دارم.					
۱۴. بیماری میاستنی زندگی من را تحت الشعاع قرار داده است.					
۱۵. من در انجام نظافت شخصی ام مشکل دارم.					

**نمره کل**	

<u>VISIT 7 (Week 48)</u>

Visit:-------

Current date:--------

Subject ID:-------------, Subject number:--------------

Enter score here:-----------------------

MG Activities of Daily Living Scale: (MG-ADL)

**Grade**	**0**	**1**	**2**	**3**	**Score (0, 1, 2, 3)**
**Talking**	Normal	Intermittent slurring of nasal speech	Constant slurring or nasal, but can be understood	Difficult to understand speech	
**Chewing**	Normal	Fatigue with solid food	Fatigue with soft food	Gastric tube	
**Swallowing**	Normal	Rare episode of choking	Frequent choking necessitating	Gastric tube	
**Breathing**	Normal	Shortness of breath with exertion	Shortness of breath at rest	Ventilator dependence	
**Impairment of ability to brush teeth or comb hair**	none	Extra effort, but no rest periods needed	Rest periods needed	Cannot do one of these functions	
**Impairment of ability to arise from a chair**	none	Mild, sometimes uses arms	Moderate, always uses arms	Severe, requires assistance	
**Double vision**	none	Occurs, but not daily	Occurs, but not daily	Constant	
**Eyelid droop**	None	Occurs, but not daily	Daily, but not constant	Constant	

Total score	

VISIT 7 (Week 48)

Visit:-------

Current date:-------- Subject ID:------------,

Subject number:-------------

Enter score here:----------------------

**MGFA Clinical Classification**	
**Class I**	Any ocular muscle weakness<br>May have weakness of eye closure<br>Strength of all the other muscles is normal
**Class II**	Mild weakness affecting muscles other than ocular muscles<br>May also have ocular muscle weakness of any severity
**IIa**	Predominantly affecting limb, axial muscles, or both.<br>May also have lesser involvement of oropharyngeal muscles
**IIb**	Predominantly affecting oropharyngeal, respiratory muscles, or both<br>May also have lesser or equal involvement of limb, axial muscles, or both
**Class III**	Moderate weakness affecting muscles other than ocular muscles<br>May also have ocular muscle weakness of any severity
**IIIa**	Predominantly affecting limb, axial muscles, or both<br>May also have lesser involvement of oropharyngeal muscles
**IIIb**	Predominantly affecting oropharyngeal, respiratory muscles, or both<br>May also have lesser or equal involvement of limb, axial muscles, or both
**Class IV**	Severe weakness affecting muscles other than ocular muscles<br>May also have ocular muscle weakness of any severity
**IVa**	Predominantly affecting limb, axial muscles, or both<br>May also have lesser involvement of oropharyngeal muscles
**IVb**	Predominantly affecting oropharyngeal, respiratory muscles, or both<br>May also have lesser or equal involvement of limb, axial muscles, or both
**Class V**	Defined as intubation, with or without mechanical ventilation, except when employed during routine postoperative management. The use of a feeding tube without intubation places the patient in class IVb.

VISIT 7 (Week 48)

Visit:-------

Current date:--------

Subject ID:------------, Subject number:-------------

The Myasthenia gravis Composite scale (MG Composite)

**Ptosis, upward (physician examination)**	$> 45\ seconds = 0$	11-45 seconds = 1	1-10 seconds = 2	Immediate = 3
**Double vision on lateral gaze, left or right (physician examination)**	$> 45\ seconds = 0$	11-45 seconds = 1	1-10 seconds = 3	Immediate = 4
**Eye closure (physician examination)**	Normal = 0	Mild weakness (can be forced open with effort) = 0	Moderate weakness (can be forced open easily) = 1	Severe weakness (unable to keep eye closed) = 2
**Talking (patient history)**	Normal = 0	Intermittent slurring or nasal speech = 2	Constant slurring or nasal but can be understood = 4	Difficult to understand speech = 6
**Chewing (patient history)**	Normal = 0	Fatigue with solid food = 2	Fatigue with solid food = 4	Gastric tube = 6
**Swallowing (patient history)**	Normal = 0	Rare episode of choking or trouble = 2	Frequent trouble swallowing e.g. necessitating changes in diet = 5	Gastric tube = 6
**Breathing (thought to be caused by MG)**	Normal = 0	Shortness of breath with exertion = 2	Shortness of breath at rest = 4	Ventilator dependence = 9
**Neck flexion or extension (weakest) (physician examination)**	Normal = 0	Mild weakness = 1	Moderate weakness (i.e. $\sim 50\% weak \pm 15\%) = 3$[a]	Severe weakness = 4
**Shoulder abduction (physician examination)**	Normal = 0	Mild weakness = 2	Moderate weakness (i.e. $\sim 50\% weak \pm 15\%) = 4$[a]	Severe weakness = 5
**Hip flexion (physician examination)**	Normal = 0	Mild weakness = 2	Moderate weakness (i.e. $\sim 50\% weak \pm 15\%) = 4$[a]	Severe weakness = 5

[a] Moderate weakness for neck and limb items should be construed as weakness that equals roughly 50% _15% of expected normal strength. Any weakness milder than that would be mild and any weakness more severe than that would be classified as severe.

Total score	

VISIT 7 (Week 48)

Please specify the MGFA Post intervention Status:

Complete Stable Remission (CSR): The patient has had no symptoms or signs of MG for at least 1 year and has received no therapy for MG during that time. There is no weakness of any muscle on careful examination by someone skilled in the evaluation of neuromuscular disease. Isolated weakness of eyelid closure is accepted.

Pharmacologic Remission (PR): The same criteria as for **CSR** except that the patient continues to take some form of therapy for MG. Patients taking cholinesterase inhibitors are excluded from this category because their use suggests the presence of weakness.

Minimal Manifestations (MM): The patient has no symptoms of functional limitations from MG but has some weakness on examination of some muscles. This class recognizes that some patients who otherwise meet the definition of **CSR** or **PR** do have weakness that is only detectable by careful examination.

MM-0: The patient has received no MG treatment for at least 1 year.

MM-1: The patient continues to receive some form of immunosuppression but no cholinesterase inhibitors or other symptomatic therapy.

MM-2: The patient has received only low-dose cholinesterase inhibitors (<120 mg pyridostigmine/day) for at least 1 year.

MM-3: The patient has received cholinesterase inhibitors or other symptomatic therapy and some form of immunosuppression during the past year.

Change in Status

Improved (I): A substantial decrease in pretreatment clinical manifestations or a sustained substantial reduction in MG medications as defined in the protocol. In prospective studies, this should be defined as a specific decrease in QMG score.

Unchanged (U): No substantial change in pretreatment clinical manifestations or reduction in MG medications as defined in the protocol. In prospective studies, this should be defined in terms of maximum change in QMG score.

Worse (W): A substantial increase in pretreatment clinical manifestations or substantial increase in MG medications as defined in the protocol. In prospective studies, this should be defined as a specific increase in QMG score.

Exacerbation (E): Patients who have fulfilled criteria of **CSR**, **PR** or **MM** but subsequently developed clinical findings greater than permitted by these criteria.

Died of MG (D of MG): Patients who died of MG, of complications of MG therapy or within 30 days after thymectomy.

VISIT 7 (Week 48)

SERIOUS ADVERSE EVENTS Page 1 of 2

Event Start Date: --------/----/---- (year/month/day), Event end Date: --------/----/---- (year/month/day)

Date Reported: --------/----/---- (year/month/day), Reported to staff by:---------------------------------

Death Date (If applicable): --------/----/---- (year/month/day)

Death Occurred (check one):

□ Within 24 hours of investigational therapy   □ Within 30 days of investigational therapy

□ Within 7 days of investigational therapy   □ After 30 days of investigational therapy

Did the SAE occur at your site or at a site for which the PI is responsible? □ Yes □No

SAE Description/Narrative:

Treating Physician Comments:

PI Comments:

Outcome: (check one)

□ Fatal/Died
□ Intervention for AE Continuing
□ Not Recovered/Not Resolved
□ Recovered/Resolved with Sequelae
□ Recovered/Resolved without Sequelae
□ Recovering/Resolving

Consent Form Change Required? □ **Yes** □ **No**

SAE Classification: (check all that apply)

□ Fatal (resulted in death)
□ A life-threatening occurrence
□ Requires inpatient hospitalization or prolongation of existing hospitalization
□ Results in persistent or significant disability/incapacity
□ Results in congenital anomaly/birth defect
□ A significant medical incident that, based upon appropriate medical judgment, may jeopardize the subject and require medical or surgical intervention to prevent one of the outcomes listed above.
□ Loss of confidentiality that results in criminal or civil liability for participation or damage to financial standing, employability, insurability or reputation of the participant

Date (dd/mm/yy): |___|___|/|___|___|/|___|___|

VISIT 7 (Week 48)

SERIOUS ADVERSE EVENTS Page 2 of 2

SAE Reported Symptom:

Category: [refer to the Safety Profiler website to search the Category and Toxicity of the SAE symptom reported: http://safetyprofiler-ctep.nci.nih.gov/CTC/CTC.aspx]

Toxicity:

Grade/Severity (check one):
□ 1 - Mild
□ 2 - Moderate
□ 3 - Severe
□ 4 - Life Threatening
□ 5 – Death (Fatal)

Unexpected? □ Yes □No

Dose Limiting Toxicity (DLT)? □ Yes □ No □ Not Applicable

Action taken:

□ Dose reduced

□ Dose interrupted, then reduced

□ None

□ Regimen interrupted

□ Therapy discontinued

Primary attribution: (check one)

□ Definite

□ Probable

□ Possible

□ Unlikely

□ Unrelated

Detailed Attribution: (check one)

□ **Disease/Condition** Specify:

□ **Investigational Treatment** Specify:

□ **Non-investigational Treatment** Specify:

□ **Other** Specify:

Date (dd/mm/yy): |___|___|/|___|___|/|___|___|

VISIT 7 (Week 48)

ADVERSE EVENT CHECK LIST

Head and Lymphatic System	Yes	No
Lymphopenia		
Leukopenia		
Neutropenia		
Thrombocytopenia		
Anemia		

Metabolic and Nutritional Disorder	Yes	No
Angioedema		
Hyperglycemia		
Peripheral Edema		

Digestive System	Yes	No
Nausea		
Diarrhea		
Vomiting		

Cardiovascular System	Yes	No
Hypotension		
Hypertension		

Musculoskeletal	Yes	No
Myalgia		
Arthralgia		

Body as a Whole	Yes	No
Fever		
Chills		
Infection		
Asthenia		
Abdominal Pain		
Pain		
Back Pain		
Throat Irritation		

Nervous System	Yes	No
Dizziness		
Anxiety		
Headache		
Progressive multifocal leukoencephalopathy		

	Yes	No
Infusion reaction		

Skin and Appendages	Yes	No
Night sweats		
Rash		
Pruritus		
Urticaria		

Date (dd/mm/yy): |___|___|/|___|___|/|___|___|

VISIT 7 (Week 48)

ADVERSE EVENTS

	**Event Name** (Please give Diagnosis if known)	**Start date** (year/month/day)	**Stop date** (year/month/day)	**Serious?** If serious, please complete a JRO SAE form	**Con-comitant Medication given**	**Severity** 0 - Mild 1- Mode-rate 2 - Severe	**Study Drug Action** 0 - None 1 - Temporarily Interrupted 2 - permanently withdrawn	**Outcome** 0 - Resolved 1- Resolved with sequelea 2 - Not resolved	**Relationship to Study Drug** 0 - Definitely 1 - Probably 2 - Possibly 3 - Unlikely 4 - Not related 5 - Not assessable
1.				□ No □ Yes	□ No □ Yes				
2.				□ No □ Yes	□ No □ Yes				
3.				□ No □ Yes	□ No □ Yes				
4.				□ No □ Yes	□ No □ Yes				
5.				□ No □ Yes	□ No □ Yes				
6.				□ No □ Yes	□ No □ Yes				
7.				□ No □ Yes	□ No □ Yes				
8.				□ No □ Yes	□ No □ Yes				
9.				□ No □ Yes	□ No □ Yes				

Date (dd/mm/yy): |___|___|/|___|___|/|___|___|

10.				□ No<br>□ Yes	□ No<br>□ Yes				
11.				□ No<br>□ Yes	□ No<br>□ Yes				
12.				□ No<br>□ Yes	□ No<br>□ Yes				
13.				□ No<br>□ Yes	□ No<br>□ Yes				
14.				□ No<br>□ Yes	□ No<br>□ Yes				
15.				□ No<br>□ Yes	□ No<br>□ Yes				
16.				□ No<br>□ Yes	□ No<br>□ Yes				
17.				□ No<br>□ Yes	□ No<br>□ Yes				
18.				□ No<br>□ Yes	□ No<br>□ Yes				

I have reviewed the AEs on this page and have assessed them for seriousness, causality, severity and outcome and confirm that, to the best of my knowledge, it accurately reflects the study information obtained for this participant

PI signature _________________________________ __________________ Date:__________________________ Please check box if this is the last page used

VISIT 7 (Week 48)

STANDARD VISIT SHEET

Visit Checklist:			
		Yes	No
1.	**Have there been any new Adverse Events?**<br>(If yes, please record in Adverse Events page)		
2.	**Have there been any changes in Concomitant Medications?**<br><br>(If yes, please record in Concomitant Medications Log)		
3.	**Laboratory data**		
4.	**Rituximab Administration**		
5.	**Is the patient experiencing a relapse?**<br><br>**(If yes record in complete physical exam form)**		
6.	**Is Adverse event check list completed?**		
7.	**Does patient withdraw or exclude from study?**		

VISIT 7 (Week 48)

PRINCIPILE INVESTIGATORE'S SIGN OFF

<table>
<tr><td colspan="2">Principal Investigator's Signature Statement:</td></tr>
<tr><td colspan="2">I have reviewed this CRF and confirm that, to the best of my knowledge, it accurately reflects the study information obtained for this participant. All entries were made either by me or by a person under my supervision who has signed the Delegation and Signature Log.</td></tr>
<tr><td>Principal Investigator's Signature:

Principal Investigator's Name:</td><td>Date of Signature:
-------/----/---- (year/month/day)</td></tr>
<tr><td colspan="2">ONCE SIGNED, NO FURTHER CHANGES CAN BE MADE TO THIS CRF WITHOUT A SIGNED DATA QUERY FORM.</td></tr>
</table>

VISIT X

MONITORING PARAMETER

Subject ID:-------------

Subject number:---------------

Name of lab that test were done?-----------------------

Date of test:-----------------

CD 19:--------------------------

CD 20:--------------------------

VISIT X

RITUXIMAB INFUSION FORM

Infusion date:------------------------------- (year, month, date)

Place of injection:--

Rituximab □ Induction phase **□ Maintenance phase**

(Please circle) week 1 2 3 4 **Month ----------------**

Dose of Rituximab administered on this infusion date:--------------

Was patient`s EKG taken before, during and after infusion?

□ Yes

□ No

If yes, please mention the condition of patient-----------------

If patient take antihypertensive drugs, did he/she hold the drug 12 hrs before rituximab infusion?

□ Yes

□ No

Use of premedication to prevent infusion reactions:

1) Was antihistamine drugs administered at this rituximab infusion?

No□

Yes□, please mention the name and dose:-------------------------

2) Was acetaminophen administered at this rituximab infusion?

No□

Yes□, please mention the dose:-------------------------

3) Was glucocorticoid drugs administered at this rituximab infusion?

No□

Yes□, please mention the name and dose:-------------------------

4) Was NSAIDs drugs administered at this rituximab infusion?

No□

Yes□, please mention the name and dose :-------------------------

VISIT X

Rituximab infusion speed protocol:

Was there infusion reaction at previous injections?

□ **Yes** :

If yes, follow this protocol: First 30 minute: 50 mg/hour (for example for 500 mg vial at 0.5 liter serum, it will be 25 ml at 30 minute that it equals 20 drops per min)

Then increased by 50 mg/hour every 30 minutes to a maximum of 400 mg/hour

□ **No:**

If no, you can follow this protocol: First 30 minute: You can start at a rate of 100 mg/hour if the initial infusion was well tolerated (for example for 500 mg vial at 0.5 liter serum, it will be 50 ml at 30 minute that it equals 40 drops per min)

Then the rate can be increased by 100 mg/hour every 30 minutes to a maximum of 400 mg/hour if there is no evidence of an infusion reaction.

Was the rituximab infusion administered completely?

□Yes □No

If No, please indicate:

Nurse name and signature:

--

VISIT X

RITUXIMAB INFUSION REACTION

Infusion Reaction:

No □ Yes □

If answer is yes, please complete details:

Time of reaction start:

□ Within 30 min

□ Within 30min-2hrs

□ After 2 hrs

□ After 24 hrs

Signs and symptoms of infusion reactions:

□ Fever and/or shaking chills
□ Flushing and/or itching
□ Alterations in heart rate and blood pressure
□ Dyspnea or chest discomfort
□ Back or abdominal pain
□ Nausea, vomiting, and/or diarrhea
□ Various types of skin rashes
□ others:--------

Classification of infusion reactions:

□ **Grade 1.** Mild transient reaction; infusion interruption not indicated; intervention not indicated

□ **Grade 2.** Therapy or infusion interruption indicated but responds promptly to symptomatic treatment (eg, antihistamines, NSAIDs, narcotics, intravenous fluids); prophylactic medication indicated for less than or equal to 24 hours

□ **Grade 3.** Prolonged (ie, not rapidly responsive to symptomatic medication and/or brief interruption of infusion); recurrence of symptoms following initial improvement; hospitalization indicated for other clinical sequelae

□ **Grade 4.** Life-threatening consequences; urgent intervention indicated

□ **Grade 5.** Death

Is anaphylactic reaction happened?

Yes □ No □

Repeated premedication after or during infusion reaction:

Yes □ No □

Treatment and dosage:

Does infusion temporarily interrupted and then continued?-----------------

Does infusion rate reduced and completed (mention the speed)?----------------

Does infusion rate reduced and not completed?--------------

<u>VISIT Y</u>

MONITORING PARAMETER

Subject ID:-------------

Subject number:---------------

Name of lab that test were done?----------------------

Date of test:-----------------

CD 19:--------------------------

CD 20:--------------------------

VISIT Y

RITUXIMAB INFUSION FORM

Infusion date:------------------------------ (year, month, date)

Place of injection:---

Rituximab □ Induction phase **□ Maintenance phase**

(Please circle) week 1 2 3 4 **Month ----------------**

Dose of Rituximab administered on this infusion date:--------------

Was patient`s EKG taken before, during and after infusion?

□ Yes

□ No

If yes, please mention the condition of patient-----------------

If patient take antihypertensive drugs, did he/she hold the drug 12 hrs before rituximab infusion?

□ Yes

□ No

Use of premedication to prevent infusion reactions:

1) Was antihistamine drugs administered at this rituximab infusion?

No□

Yes□, please mention the name and dose:-------------------------

2) Was acetaminophen administered at this rituximab infusion?

No□

Yes□, please mention the dose:------------------------

3) Was glucocorticoid drugs administered at this rituximab infusion?

No□

Yes□, please mention the name and dose:-------------------------

4) Was NSAIDs drugs administered at this rituximab infusion?

No□

Yes□, please mention the name and dose :-------------------------

VISIT Y

Rituximab infusion speed protocol:

Was there infusion reaction at previous injections?

□ **Yes** :

If yes, follow this protocol: First 30 minute: 50 mg/hour (for example for 500 mg vial at 0.5 liter serum, it will be 25 ml at 30 minute that it equals 20 drops per min)

Then increased by 50 mg/hour every 30 minutes to a maximum of 400 mg/hour

□ **No:**

If no, you can follow this protocol: First 30 minute: You can start at a rate of 100 mg/hour if the initial infusion was well tolerated (for example for 500 mg vial at 0.5 liter serum, it will be 50 ml at 30 minute that it equals 40 drops per min)

Then the rate can be increased by 100 mg/hour every 30 minutes to a maximum of 400 mg/hour if there is no evidence of an infusion reaction.

Was the rituximab infusion administered completely:

□**Yes** □**No**

If No, please indicate:

Nurse name and signature:

--

VISIT Y

RITUXIMAB INFUSION REACTION

Infusion Reaction:

No □ Yes □

If answer is yes, please complete details:

Time of reaction start:

□ Within 30 min

□ Within 30min-2hrs

□ After 2 hrs

□ After 24 hrs

Signs and symptoms of infusion reactions:

□ Fever and/or shaking chills
□ Flushing and/or itching
□ Alterations in heart rate and blood pressure
□ Dyspnea or chest discomfort
□ Back or abdominal pain
□ Nausea, vomiting, and/or diarrhea
□ Various types of skin rashes
□ others:--------

Classification of infusion reactions:

□ **Grade 1.** Mild transient reaction; infusion interruption not indicated; intervention not indicated

□ **Grade 2.** Therapy or infusion interruption indicated but responds promptly to symptomatic treatment (eg, antihistamines, NSAIDs, narcotics, intravenous fluids); prophylactic medication indicated for less than or equal to 24 hours

□ **Grade 3.** Prolonged (ie, not rapidly responsive to symptomatic medication and/or brief interruption of infusion); recurrence of symptoms following initial improvement; hospitalization indicated for other clinical sequelae

□ **Grade 4.** Life-threatening consequences; urgent intervention indicated

□ **Grade 5.** Death

Is anaphylactic reaction happened?

Yes □ No □

Repeated premedication after or during infusion reaction:

Yes □ No □

Treatment and dosage:

Does infusion temporarily interrupted and then continued?----------------

Does infusion rate reduced and completed (mention the speed)?---------------

Does infusion rate reduced and not completed?--------------

VISIT Z

MONITORING PARAMETER

Subject ID:-------------

Subject number:---------------

Name of lab that test were done?----------------------

Date of test:-----------------

CD 19:--------------------------

CD 20:--------------------------

VISIT Z

RITUXIMAB INFUSION FORM

Infusion date:------------------------------ (year, month, date)

Place of injection:---

Rituximab □ Induction phase **□ Maintenance phase**

(Please circle) week 1 2 3 4 **Month ----------------**

Dose of Rituximab administered on this infusion date:--------------

Was patient`s EKG taken before, during and after infusion?

□ Yes

□ No

If yes, please mention the condition of patient-----------------

If patient take antihypertensive drugs, did he/she hold the drug 12 hrs before rituximab infusion?

□ Yes

□ No

Use of premedication to prevent infusion reactions:

1) Was antihistamine drugs administered at this rituximab infusion?

No□

Yes□, please mention the name and dose:-------------------------

2) Was acetaminophen administered at this rituximab infusion?

No□

Yes□, please mention the dose:-------------------------

3) Was glucocorticoid drugs administered at this rituximab infusion?

No□

Yes□, please mention the name and dose:-------------------------

4) Was NSAIDs drugs administered at this rituximab infusion?

No□

Yes□, please mention the name and dose :-------------------------

VISIT Z

Rituximab infusion speed protocol:

Was there infusion reaction at previous injections?

□ **Yes** :

If yes, follow this protocol: First 30 minute: 50 mg/hour (for example for 500 mg vial at 0.5 liter serum, it will be 25 ml at 30 minute that it equals 20 drops per min)

Then increased by 50 mg/hour every 30 minutes to a maximum of 400 mg/hour

□ **No:**

If no, you can follow this protocol: First 30 minute: You can start at a rate of 100 mg/hour if the initial infusion was well tolerated (for example for 500 mg vial at 0.5 liter serum, it will be 50 ml at 30 minute that it equals 40 drops per min)

Then the rate can be increased by 100 mg/hour every 30 minutes to a maximum of 400 mg/hour if there is no evidence of an infusion reaction.

Was the rituximab infusion administered completely:

□**Yes** □**No**

If No, please indicate:

Nurse name and signature:

VISIT Z

RITUXIMAB INFUSION REACTION

Infusion Reaction:

No □ Yes □

If answer is yes, please complete details:

Time of reaction start:

□ Within 30 min

□ Within 30min-2hrs

□ After 2 hrs

□ After 24 hrs

Signs and symptoms of infusion reactions:

□ Fever and/or shaking chills
□ Flushing and/or itching
□ Alterations in heart rate and blood pressure
□ Dyspnea or chest discomfort
□ Back or abdominal pain
□ Nausea, vomiting, and/or diarrhea
□ Various types of skin rashes
□ others:--------

Classification of infusion reactions:

□ **Grade 1.** Mild transient reaction; infusion interruption not indicated; intervention not indicated

□ **Grade 2.** Therapy or infusion interruption indicated but responds promptly to symptomatic treatment (eg, antihistamines, NSAIDs, narcotics, intravenous fluids); prophylactic medication indicated for less than or equal to 24 hours

□ **Grade 3.** Prolonged (ie, not rapidly responsive to symptomatic medication and/or brief interruption of infusion); recurrence of symptoms following initial improvement; hospitalization indicated for other clinical sequelae

□ **Grade 4.** Life-threatening consequences; urgent intervention indicated

□ **Grade 5.** Death

Is anaphylactic reaction happened?

Yes □ No □

Repeated premedication after or during infusion reaction:

Yes □ No □

Treatment and dosage:

Does infusion temporarily interrupted and then continued?----------------

Does infusion rate reduced and completed (mention the speed)?---------------

Does infusion rate reduced and not completed?--------------
